# Inference of Functionally-Relevant N-acetyltransferase Residues Based on Statistical Correlations

**DOI:** 10.1371/journal.pcbi.1005294

**Published:** 2016-12-21

**Authors:** Andrew F. Neuwald, Stephen F. Altschul

**Affiliations:** 1 Institute for Genome Sciences and Department of Biochemistry & Molecular Biology, University of Maryland School of Medicine, BioPark II, Room 617, Baltimore, MD, United States of America; 2 National Center for Biotechnology Information, National Library of Medicine, National Institutes of Health, Bethesda, MD, United States of America; Center for Cancer Research, UNITED KINGDOM

## Abstract

Over evolutionary time, members of a superfamily of homologous proteins sharing a common structural core diverge into subgroups filling various functional niches. At the sequence level, such divergence appears as correlations that arise from residue patterns distinct to each subgroup. Such a superfamily may be viewed as a population of sequences corresponding to a complex, high-dimensional probability distribution. Here we model this distribution as hierarchical interrelated hidden Markov models (hiHMMs), which describe these sequence correlations implicitly. By characterizing such correlations one may hope to obtain information regarding functionally-relevant properties that have thus far evaded detection. To do so, we infer a hiHMM distribution from sequence data using Bayes’ theorem and **Markov chain Monte Carlo (MCMC)** sampling, which is widely recognized as the most effective approach for characterizing a complex, high dimensional distribution. Other routines then map correlated residue patterns to available structures with a view to hypothesis generation. When applied to N-acetyltransferases, this reveals sequence and structural features indicative of functionally important, yet generally unknown biochemical properties. Even for sets of proteins for which nothing is known beyond unannotated sequences and structures, this can lead to helpful insights. We describe, for example, a putative coenzyme-A-induced-fit substrate binding mechanism mediated by arginine residue switching between salt bridge and π-π stacking interactions. A suite of programs implementing this approach is available (psed.igs.umaryland.edu).

## Author Summary

Protein sequence data, when gathered in great quantity, contain important but implicit biological information manifest as statistical correlations. Here we describe an approach to access this information by comprehensively modeling and characterizing the distribution of sequences belonging to a major protein superfamily. This approach takes as input a large set of unaligned sequences belonging to the superfamily. By applying the minimum description length principle, it seeks the statistical model that best explains the sequences while avoiding over-fitting the data. It concurrently aligns the sequences and, to model evolutionary divergence, partitions them into subgroups that are hierarchically-arranged based upon correlated residue patterns. Auxiliary routines create PyMOL scripts to visualize the locations of correlated residues within available structures. Because these correlations likely arise from structural and biochemical constraints, they can help elucidate protein properties important for functional specificity. Comparing and contrasting sequence and structural features in this way may therefore suggest, in the light of published studies, plausible biological hypotheses for experimental investigation. We illustrate this approach with N-acetyltransferases.

## Introduction

**Fig 1:**
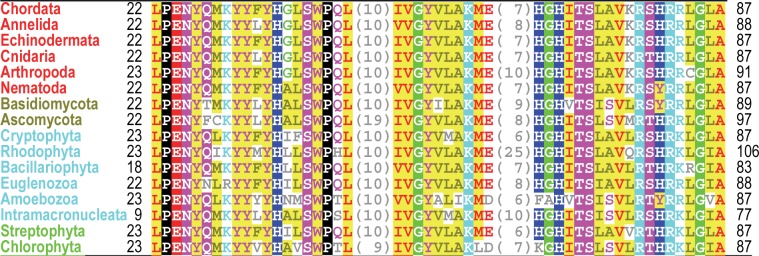
Residues, mostly of unknown function, that are highly conserved in putative orthologs of human Naa10 acetylase from metazoans (phyla indicated in red), fungi (brown), protozoans (cyan), and plants (green).

Mendel used statistics to infer the existence of genes [1], and other early geneticists to infer their linear arrangement when not independently assorted, thereby recognizing biological phenomena whose precise physical nature was illuminated only later. Statistics can continue to play an important role in biological discovery. Specific residues within homologous proteins are often conserved over long evolutionary time and across diverse organisms. For instance, putative orthologs of the NatA N-acetyltransferase catalytic subunit Naa10 from human (**Fig 1**) [2] contain many invariant or highly-conserved residues; indeed, natural selection has sometimes eliminated even the rearrangement of a side-chain methyl group or the removal of a hydroxyl group. Such conservation implies the participation of these protein residue positions in biologically critical functions or interactions. The steric or chemical constraints giving rise to this conservation are sometimes understood, but often the conservation remains unexplained, implying protein properties and functions that are still to be discovered. Moreover, many residues are conserved across different acetyltransferase families, which are associated with distinct cellular complexes, and which vary widely in their localization, turnover, inter-protein interactions and binding kinetics. Hence the roles of these residues presumably transcend functions specific to individual families and instead may involve multi-purpose mechanisms or interactions shared by otherwise functionally-distinct proteins. Similar comments also apply to many other protein superfamilies—defined as homologous proteins sharing detectable (albeit possibly very subtle) sequence similarity and a common core structure.

### Protein statistical inferences

Just as one may infer genetic information from phenotypic patterns among parents and offspring, so one may infer biochemical and structural information from patterns of amino acid conservation and divergence among related proteins. In either case, careful statistical analyses are of course essential to ensure the reliability of results, as serious errors in biomedical studies illustrate [3, 4]. The inference of protein structural information has been illustrated recently through predictions using covariance analysis of **multiple sequence alignments (MSAs)** [5–7]. Although MSA covariance analyses had been performed previously, key breakthroughs only came through the application of more sophisticated approaches, such as Direct Coupling Analysis [8] and the pseudo-likelihood method [9]. An important contribution to DCA’s success is the avoidance of over-fitting using maximum entropy probability models [10].

### Functionally-relevant correlated residues

Rather than focusing on correlated residue pairs using a covariance matrix, our focus is on correlations more loosely defined as dependencies involving many residue positions. In particular, we seek to identify arbitrarily-long correlated residue patterns, which we hypothesize as due to protein functional divergence. Over evolutionary time members of a major protein superfamily diverge into subgroups, each of which fills a functional niche compatible with the common “core" structure defining the superfamily. Often a subgroup, *G*, is composed of smaller subgroups, each of which conserves both residues due to *G*’s functional constraints and other residues due to constraints imposed by its own, more specialized function. Repeated rounds of this evolutionary process have led to hierarchically-interrelated patterns of correlated residues. As a result, members of a protein superfamily tend to cluster into multiple subgroups where each subgroup tends to conserve distinctive residues at particular sequence positions. One consequence of this is that different amino acid residues may be conserved at the same positions among distinct subgroups. Our focus here is on defining, based on these residue patterns, a hierarchically nested series of multiple sequence alignments corresponding to such subgroups. Modeling correlations through the articulation of subgroups within a protein superfamily in this way provides an alternative formalism to the standard practice of using a covariance matrix to describe explicit correlations between pairs of positions (e.g., [5–7]). Note that our model does not assume any correlation between residues within a subgroup.

### The hiMSA sampler

Previously [11–19] we have used Bayesian **Markov chain Monte Carlo (MCMC)** sampling [20] to identify correlated residue patterns within a (static) MSA with a view to obtaining clues to underlying protein properties. This approach, termed **Bayesian Partitioning with Pattern Selection (BPPS)** [21–24], models correlated residues indirectly by hierarchically partitioning a MSA into subgroups based on residue patterns distinguishing each subgroup, *G*, from other subgroups comprising the next-higher-level subgroup to which *G* belongs. Here we extend this approach by sampling over both alignments and hierarchies—that is, over possible **hierarchical interrelated MSAs (hiMSAs)** and over corresponding **hierarchical interrelated hidden Markov models (hiHMMs).** We use simulated annealing [25] to facilitate convergence on the hiHMM most likely to have generated the input sequences; in this study, those belonging to the N-acetyltransferase superfamily. To ensure that pattern residues are due to persistent functional constraints rather than recent common descent, we focus on residues conserved across distinct phyla. We define “functional constraints” to include significant selective advantages imposed by *any* protein property, including catalytic and substrate-binding properties, other binding sites, folding and structural properties, and protein stability.

### FDR methods

Some methods align and classify protein sequences concurrently [26–32] and a wide variety of methods aim to identify protein **function determining residues** (**FDRs**) [33–53] However, we are unaware of a method that aligns and classifies sequences and identifies FDRs concurrently. Moreover, FDR methods differ from hiMSA sampling in four respects: (1) FDR methods generally focus on predicting specific, well-characterized residue functions, such as in catalysis and substrate recognition, that can be experimentally benchmarked [54]. However, as noted recently [55, 56], we lack reliable gold standards due to the incompleteness of experimental annotations. Consequently, methods meeting these standards will score correlated residues involved in important but uncharacterized functions incorrectly as false positives and thus will fail to characterize correlated residues objectively, which is our goal here. (2) Many cannot handle large, diverse data sets and therefore cannot adequately model a major protein superfamily. Typically this limitation arises from focusing on residue changes within a phylogenetic tree, which, for hundreds of thousands of sequences, introduces more complexity than either is necessary or can be reliably inferred. Instead, the hiMSA sampler models a protein superfamily as a simpler hierarchy of related subgroups, each of which is defined by a correlated residue pattern (and presumably by corresponding protein properties). (3) Most FDR methods lack a rigorous statistical basis and thus a way to distinguish signals from noise and to assess significance. Doing so requires adjusting for the implicit number of alternative hypotheses considered during optimization, which is not trivial given real-valued parameters and the vastness of the search space. (4) FDR methods typically depend upon pre-computed input (e.g., a tree or alignment) that has been optimized using an objective function that is distinct from and often statistically inconsistent with that of the method itself. Moreover, phylogenetic and clustering programs will fail to adequately distinguish signals from noise insofar as they rely on conventional MSA programs (e.g., [57–62]), nearly all of which will align random sequences and that therefore over-fit the data.

### Advantages of hiMSA sampling

The hiMSA sampler constitutes a significant shift from existing methods. **(1)** It concurrently, comprehensively, and in a statistically coherent manner defines correlated residue patterns, corresponding hierarchically-arranged (and presumably functionally-divergent) sequence subgroups, and the extent of each subgroup's alignment. None of these elements are known *a priori*, and knowledge of each contributes to the proper recognition of the others. For example, each divergent subgroup needs to be defined and properly aligned to identify correlated residue patterns; however, such patterns are required to define each subgroup. Our approach avoids this chicken or egg dilemma by iteratively sampling each element in turn. **(2)** We find that MCMC sampling often exhibits a favorable time complexity [63], making it applicable even to very large data sets (e.g., 424,764 sequences in [64]). Finding an optimal or near-optimal hiHMM using maximum-likelihood as a criterion requires searching a very high-dimensional and complex space. Although rigorous optimization is intractable, Bayesian MCMC sampling [20] is widely recognized as the most effective method for locating near-optimal solutions in such circumstances. And, unlike deterministic methods, which may repeatedly return the same suboptimal result with no indication of uncertainty, MCMC sampling can reveal uncertainties and thereby the degree to which modeled features and parameters may be trusted. **(3)** It avoids modeling extraneous features and random noise by applying the **minimum description length (MDL)** principle [65], which provides a criterion for choosing among alternative models for describing a set of data. Conceptually, it suggests that the best model, among a set of alternatives, is that which minimizes the description length of the model, plus the maximum-likelihood description length of the data given the model. This adjusts for the implicit number of hypotheses considered while exploring possible models—thereby ensuring that sequence regions are aligned and that correlated residue patterns and corresponding subgroups are defined *only* when justified statistically. As is illustrated here, in the light of the literature and of available structural data [66], enhancing signal over noise in this way aids biological interpretation of hiMSA properties. **(4)** Finally, it explicitly models non-sub-classifiable sequences and identifies unrelated or aberrant sequences that may have been included inadvertently.

## Results

**Fig 2:**
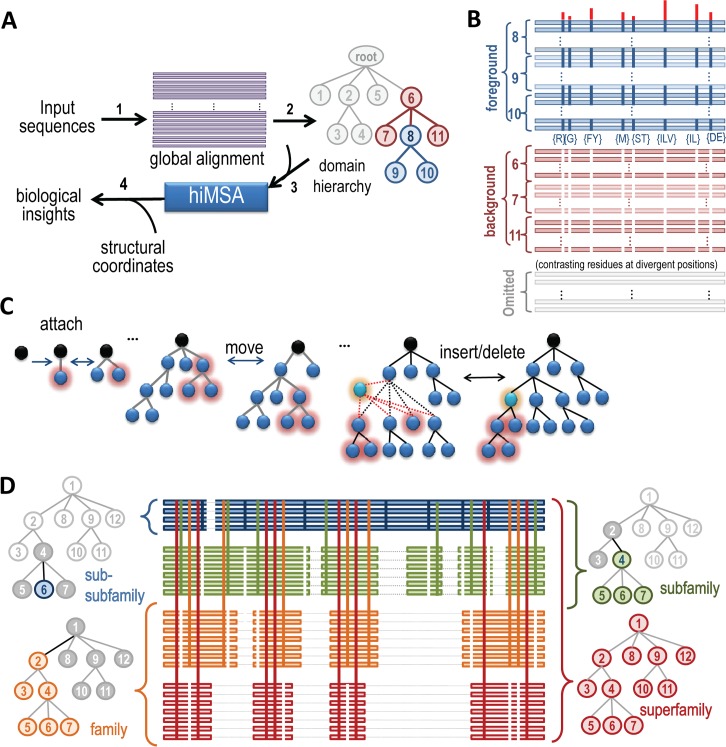
hiMSA creation and analysis. **A**. Flow chart showing the steps required to create and interpret a hiMSA (as described in text). **B**. Schematic of a BPPS-generated “**contrast alignment**” that corresponds to node 8 of the hierarchy in (A). One such contrast alignment is created for each node in the hierarchy. Sequences assigned to node 8’s subtree (blue nodes in (A)) constitute a ‘foreground’ partition, those assigned to the most closely related nodes (red nodes in (A)) constitute a ‘background’ partition, and the remaining sequences constitute a non-participating partition. Horizontal bars represent sequences assigned to the similarly-colored corresponding nodes in (A). Blue vertical bars represent conserved foreground residue patterns (as shown below each bar); these diverge from (or contrast with) the background compositions at those positions (white vertical bars). Red vertical bars above the alignment quantify the degree of divergence. **C**. BPPS sampling explores the space of domain hierarchies by attaching or removing leaf nodes, moving subtrees, inserting or deleting internal nodes, moving sequences between nodes and, for each subtree, adding or deleting residue patterns based on how well they discriminate the foreground from the background (as shown in (B)). **D**. Schematic diagram of a hiMSA from the perspective of a leaf node. One such diagram could be created for each node in a hierarchy. (*center*) The node 6 lineage of the full hiMSA. Horizontal lines represent aligned sequences and are color-coded by level in the hierarchy. Thin light gray horizontal lines represent non-homologous and deleted regions. Vertical lines represent the contrasting pattern positions upon which the hierarchy is based and are similarly color-coded by levels. (*left & right sides*) Subtrees corresponding to each level. The colored, gray and white nodes in each tree correspond, respectively, to their alignment foreground, background and non-participating partitions, the sequences of which are colored similarly. The background for the entire superfamily (lower right) consists of random sequences.

**Fig 2A** shows the specific steps used to obtain and analyze a hiMSA: **(1)** Search for and align database sequences belonging to a protein domain superfamily using MAPGAPS [67], which takes as the query either a curated MSA of representative sequences or an existing hiMSA; the MAPGAPS-generated MSA is improved via MCMC sampling [68–71] using GISMO [63]. **(2)** Partition the alignment into hierarchically-arranged subgroups based on correlated residue patterns using BPPS [21–24] (**Fig 2B and 2C**). **(3)** Using the hieraln program (first described here) create a hiMSA. **(4)** Visualize hiMSA-associated sequence and structural properties using the hierview program (first described here), and correlate these properties with other biological information to obtain clues to protein function as an aid to experimental design.

### MSA sampling

In step 1 of **Fig 2A**, GISMO [63] improves the initial MSA by sampling over a **hidden Markov Model (HMM)** posterior probability distribution given the input sequence data and Dirichlet mixture priors [72–74]. An HMM [75] is a generative statistical model that, as applied here, defines a distribution of protein sequences. It can generate sequences by sampling paths between its start and end states (**S1.1A Fig**), while “emitting” an amino acid residue for each match and insert state encountered along each path. Corresponding to an HMM-generated set of sequences is an MSA that defines the path taken for each sequence, where match and delete states correspond to aligned columns and insert states to insertions. Conversely, from an MSA may be inferred the states and the emission and transition probabilities for a corresponding HMM. GISMO improves an MSA by iterating between inferring a new HMM given a current MSA and sampling a new MSA given a current HMM. It similarly infers position-specific transition probabilities based on the transitions implied by the MSA. Given a sufficiently large number of input sequences, an accurate MSA and HMM for a protein superfamily may be inferred in this way.

### BPPS sampling

In Step 2 of **Fig 2A**, the BPPS sampler partitions the MSA into hierarchically-arranged subgroups based on correlated residues distinctive of each subgroup (**Fig 2B**). Starting from a single node, it samples new child nodes (each after a period of burn-in sampling that allows the Markov chain to approach its steady-state). This operation and ‘move’, ‘insert’ and ‘delete’ operations (**Fig 2C**) are sampled iteratively to explore possible hierarchies. Each hierarchy, in conjunction with specific sequence and pattern assignments, is sampled with probability proportional to the degree to which its pattern residues are more highly conserved in ‘foreground’ versus ‘background’ sequences (**Fig 2B**)—collectively, over all nodes in the hierarchy. Note that the probability distribution (as defined in Eq 4 of Methods) treats each column position as statistically independent; hence BPPS models correlations indirectly, based on the hierarchy. **S5 Fig** illustrates how BPPS sampling produces hierarchies that, based on measures of statistical significance, are generally superior to hierarchies created at the NCBI using a combination of phylogenetic analysis and manual-curation; for in-depth evaluations of protein domain hierarchies in this way, see [23, 24, 76].

### The hiMSA sampler

A simple MSA and corresponding HMM fails to model a major protein superfamily adequately due to insertions and deletions associated with functional divergence and specialization. Instead we model a superfamily as a hiHMM corresponding to a hiMSA (**Fig 2D**), and optimize it using a two-component hiMSA sampler (Step 3 in **Fig 2A**), as follows. The BPPS component partitions a simple (non-hierarchical) alignment into hierarchically-arranged subgroups (**Fig 2C**) based upon patterns of conserved and divergent residues (**Fig 2B**), while the MSA component adjusts the sub-alignment associated with each subgroup to align regions conserved in the subgroup but not in the superfamily as a whole, thereby creating a hiMSA (step 3 of **Fig 2A**). Corresponding to this hiMSA is a hiHMM, which is defined as follows: A HMM is defined for each subgroup (i.e., subtree in the hierarchy) with **lineage-specific emission probabilities** for each match state (i.e., sub-alignment column). To understand what this entails, consider the lineage shown in **Fig 2D**: For a given node (e.g., node 6 in this figure), the hiMSA sampler assigns columns either to that node (blue columns), or to another node along the path back to the root (e.g., green, orange or red columns). For the HMM associated with leaf node 6, the emission probabilities corresponding to blue columns are inferred based solely on node-6-assigned sequences. The rationale for this is that the residue distributions for these column positions diverge from the distributions of the parental node. Likewise, the emission probabilities corresponding to green, orange and red discriminating columns in **Fig 2D** are inferred based solely on the sequences assigned, respectively, to subtrees rooted at nodes 4, 2 and 1 (see **S1.1C Fig**). In general, for a discriminating column *C* of node *N* the corresponding emission probabilities are based on all of the residues in that column of the subtree rooted at *N*. Finally, each of the HMMs within the hiHMM is assigned a weight proportional to the fraction of sequences (after down-weighting for redundancy [77]) assigned to each of the corresponding nodes. Together, these interrelated HMMs collectively define a hiHMM and the maximum-likelihood hiMSA, which we seek. To be biologically most useful the hiMSA sampler should find the hiHMM most likely to have generated the input sequences for a given protein domain superfamily. This, in turn, requires that the MSA and BPPS models be combined into a single, statistically-coherent model, that efficient strategies be devised for hiHMM sampling with simulated annealing, and that sampling be done over a sufficiently large sequence set.

### The MDL principle

The **minimum description length (MDL)** principle [65] provides criteria for combining the HMM and BPPS models into a single model and for choosing among alternative model architectures and parameters describing the sequence data. Conceptually, it suggests that the best model, among a set of alternatives, is that which minimizes the description length of the model, plus the maximum-likelihood description length of the data given the model. This penalizes the freedom that the sampler has to choose among model architectures and their parameters. In the context of this study, the MDL principle allows us to balance the improved prediction of residue frequencies that arises from the articulation of protein subgroups with the increased model complexity that such articulation implies. While the MDL principle allows homologous residues to be aligned at discriminating positions, it disfavors the unwarranted generation of merely artificial patterns and subgroups. This is analogous to ensuring that a MSA procedure both adds gaps to align homologous residues and avoids adding gaps to align similar but non-homologous residues. In this way we can ensure that increases in hiHMM architectural complexity through the alignment of dissimilar residues occurs only when justified statistically.

From an information theoretical perspective, applying the MDL principle to our hiHMM can be thought of as follows. Imagine that we seek to transmit 1,000 input sequences, each of which is 100 residues long, over a communication channel using a binary code. If the sequences are entirely random, then the most efficient way to do this is to base our coding upon the overall residue composition of the sequences. This would correspond to a null model consisting of a single insert state, where, for example, the most and least common residues use the shortest and longest bit strings, respectively. However, if the sequences are related over their entire lengths, we can use a more efficient coding based on a simple (100 match state) HMM that uses the optimal coding at each sequence position. In this case, however, we also need a longer description for the HMM. Doing this is justified only if the increased description length of the more complex model is offset by the decreased length of the transmitted sequence data.

### The MDL principle and simple HMMs

The MDL principle may be applied either in its information theoretical form just described or, alternatively, using a Bayesian approach that defines prior probabilities for the null and each of the possible non-null HMM architectures (i.e., possible number of states and thus of aligned columns in the corresponding MSA). To illustrate this approach, we first consider the simplest case of MCMC sampling over possible architectures for a single (non-hierarchical) HMM (**S1.1A Fig**). In this case our goal is to best fit the HMM's architecture to a set of input sequences. The simplest such architecture corresponds to the null HMM consisting of a single insert state. More complicated architectures add additional sets of match, delete and insert states, so the architectures differ only in the total number of such sets. At its most elementary, selecting an architecture thus involves selecting a non-negative integer. Because we place no bound on this integer, we need to assign positive priors to the possible architectures that sum to one. A simple geometric series suffices, and choosing a geometric constant near one can render us essentially indifferent among architecture sizes. The MDL principle has its main effect when the input data must be fit to the various architectures.

Here, each additional set of states requires, for each input sequence, one additional choice in describing its path through the HMM. The information required to specify these choices can be offset only by the prediction of residue frequencies in a new match state that are sufficiently improved vis a vis an insert state. Other aspects of an optimal alignment remaining fixed, this is achieved only when the Bayesian Integral Log-odds (BILD) score [78] of the newly aligned residues is positive. The BILD score for an MSA column, $\vec{x}$, is the log-odds ratio for observing $\vec{x}$ under two alternative hypotheses [78], *H*_*1*_ and *H*_*2*_. Here, *H*_*1*_ is a Dirichlet mixture model for related proteins [72–74] and *H*_*2*_ is the null model of unrelated proteins.

### The MDL principle and hiHMMs

The complex, hierarchical structure of dependencies among most large protein families provides further opportunity for the reduction of total data description length using the MDL principle. In brief, sequence divergence at various positions among different protein subgroups coordinated with sequence conservation within each subgroup permits a hiHMM to describe the sequence data more compactly by tailoring the amino acid emission probabilities of HMM match states to each individual subgroup. This comes at the cost of the increased complexity, and thus description length, of hiHMMs, and the MDL principle allows us to balance these competing considerations. We consider here, in increasing order, the contributions of various hiHMM elements to the total description length: the number of nodes in the hiHMM hierarchy; the rooted tree defining this hierarchy; the discriminating columns for each node and their corresponding residue sets; the sequences assigned to each node; the number of states in each of the hierarchically-arranged HMMs, and the sequences' paths through these states. We will illustrate each of these description lengths with a specific example, and consider how the description lengths can be offset by a shorter description length for the sequence data.

First, the number of nodes in the hierarchy is again just a positive number. As before we can assign to these numbers prior probabilities that sum to one based on a slowly decaying geometric series, thereby rendering the selection of the number of nodes, *n*, nearly irrelevant to the total description length. Specifically, if we specify a geometric factor *x*, the description length of *n* will be −log_2_(1−*x*)−(*n*−1)log_2_*x* bits. For example, if we choose *x* = 0.99, the description length of *n* = 1 would be approximately 6.6 bits, and for *n* = 250 would be about 10.2 bits, which as we will see is insignificant.

Second, the number of possible rooted trees on *n* nodes increases exponentially with *n*. Specifically, the number of distinct rooted, unlabeled (i.e., non-isomorphic, *n*-ary) trees on *n* nodes is given by the Catalan number [79]

$$C_{n}=\frac{1}{n+1}\left(\begin{array}{c} 2n\\ n \end{array}\right)=\frac{\left(2n\right)!}{\left(n+1\right)!n!}.$$

For a given *n*, we can assign all these trees equal prior probability. Asymptotically, the Catalan numbers are approximately [80]

$$C_{n}\approx \frac{4^{n}}{n^{3/2}\sqrt{\pi }},$$

so that the description length of a specific tree is 2*n*−1.5log_2_*n*−0.6 bits, or roughly 2*n* bits. For example the description length of a specific tree having *n* = 250 nodes is about 487.5 bits. As we will see, compared to other elements in the description length of a hiHMM model, this is again essentially insignificant. In practice, for both algorithmic and biological reasons, we limit the trees we consider to those having a height ≤ *h*, where *h* = 5 by default.

Third, for each node, we must describe the number of discriminating columns, and the residue set associated with each column. We allow only residues sets that have biologically sensible patterns, and further constrain the set specified for a column to include the consensus residue among the sequences aligned in that column. Let us assign a prior probability *p* to a column being discriminating, and assume there are about *t* allowable and equally probable residue patterns at each column, and *c* total columns in the HMM for a given node. Then the description length of the discriminating pattern for the node is approximately *c*[−(1−*p*)log_2_(1−*p*)−*p*log_2_(*p*/*t*)] bits. For example, if *c* = 200, *t* = 10, and we choose *p* = 0.1, this comes to about 160 bits per node. For *n* = 250 nodes, the description length of all discriminating columns and their associated patterns is about 40,000 bits. In practice, we limit the number of discriminating columns for each node to ≤ *m*, where *m* = 25 by default, and place decreasing prior probabilities on progressively larger residue sets, as previously described [22].

Fourth, we must assign each sequence to a node. Assuming all *n* nodes are equally probable, this requires log_2_*n* bits per sequence. For *n* = 250, this is about 8 bits per sequence and for 25,000 effectively independent sequences (i.e., after down-weighting for redundancy), about 200,000 bits. In practice, to avoid excessively elaborate hierarchies, we require by default that at least 50 sequences be assigned to each leaf node and that each node contribute at least 7 bits of information.

The total description of the specific hiHMM considered above is approximately 240,000 bits. To justify such a complex model, the description length of the data requires an equivalent improvement. We have assumed a core alignment of *c* = 200 columns, and *s* = 25,000 sequences, which implies 5,000,000 residues in the core. If the average description length of these residues is decreased by over 0.048 bits/residue, it will justify the increased complexity of the model.

Finally, we have ignored so far that we may adjust the HMM architecture for each node. Any extra HMM states at a given node will require the description of the paths of the sequences assigned to that node or its descendants through the HMM, but this increased description length can be offset by positive BILD scores for the newly aligned residues. (Since the maximum likelihood parameter settings for the hiHMM are inferred from the hiMSA, these are implicitly defined by the hiMSA—that is, by each aligned sequence’s path through the HMM for the sequence’s assigned node. Therefore, we merely need to determine the MDL of the hiMSA instead of the hiHMM. This involves integrating out the hiHMM’s parameters given the hiMSA using a single set of fixed priors for hiHMM emission and transition probabilities. We recently applied the MDL principle in this way to an MSA [63], generalization of which to a hiMSA is straightforward.)

This basic approach may be modified in various ways. For example, the prior probabilities for the node assignments of individual sequences need not be equal, and may be defined to sum to 1 in various ways. To favor shallow hierarchies, a high prior probability may be assigned to the root node, and progressively lower probabilities to nodes lower in the hierarchy. To favor deeper hierarchies, all nodes (except perhaps the root) may be assigned equal prior probabilities. By default, we set the prior probability for the root node to 0.25, for a “reject node” to 0.70, and equally for each of the remaining nodes so as to sum to 0.05. The reject node corresponds to unrelated and thus unalignable sequences that lack features of the entire superfamily and that presumably were included inadvertently. Note that, given an input set of unrelated sequences, the MDL principle, as just applied, favors the “null hierarchy” consisting solely of this reject node.

### Sampling strategies

To search for a hiHMM optimally modeling the distribution implied by a large set of input sequences, our samplers do the following: **(1)** Construct an MSA using the GISMO sampler [63]. To speed up sampling over very large sequence sets, one may first align a representative subset and then sample in the remaining sequences after convergence. **(2)** Next the following substeps are performed (in arbitrary order) until the evolving hiHMM fails to improve after a specified number of cycles: **(2.a)** Nodes are sampled in and out of the hierarchy as illustrated in **Fig 2C**. **(2.b)** Discriminating patterns are sampled for each node. **(2.c)** Sequences are sampled between nodes. **(3)** Starting with each child node of the root and proceeding recursively to descendent nodes, the extent of each node’s sequence alignment is adjusted using the GISMO sampler [63] to include subgroup-specific regions (**S1.2 Fig**). This generates the hiMSA. **(4)** Substep 2.b is applied to the hiMSA to identify correlated residue patterns within subgroup-specific regions. We use BPPS sampling to define the hierarchy based on those residue patterns that most discriminate between divergent subgroups; BILD scores [78] to assign aligned columns to specific nodes in the hierarchy; Dirichlet mixture priors [72–74] to accommodate small sequence sets; down-weighting for sequence redundancy [77, 81]; and, after convergence, simulated annealing [25] to drop into an (ideally nearly global) optimum. In principle, finding the global optimum for all but the simplest hierarchies is nearly impossible. In practice, however, we find that the various hierarchies obtained from run to run generally share the biologically most important features of the superfamily [76] and that the features most difficult to model optimally are least important. Therefore, failing to find the global optimum often forfeits little of biological significance.

### Obtaining biological insight

The final step in **Fig 2A** is to interpret the results biologically. This involves: (i) identifying and characterizing putative sequence determinants of subgroup-specific functions; (ii) interpreting these in the light of available structures and other biological information; and (iii) formulating hypotheses for experimental follow up. We illustrate this here for N-acetyltransferases.

## Application

### N-acetyltransferases (acetylases)

**Fig 3:**
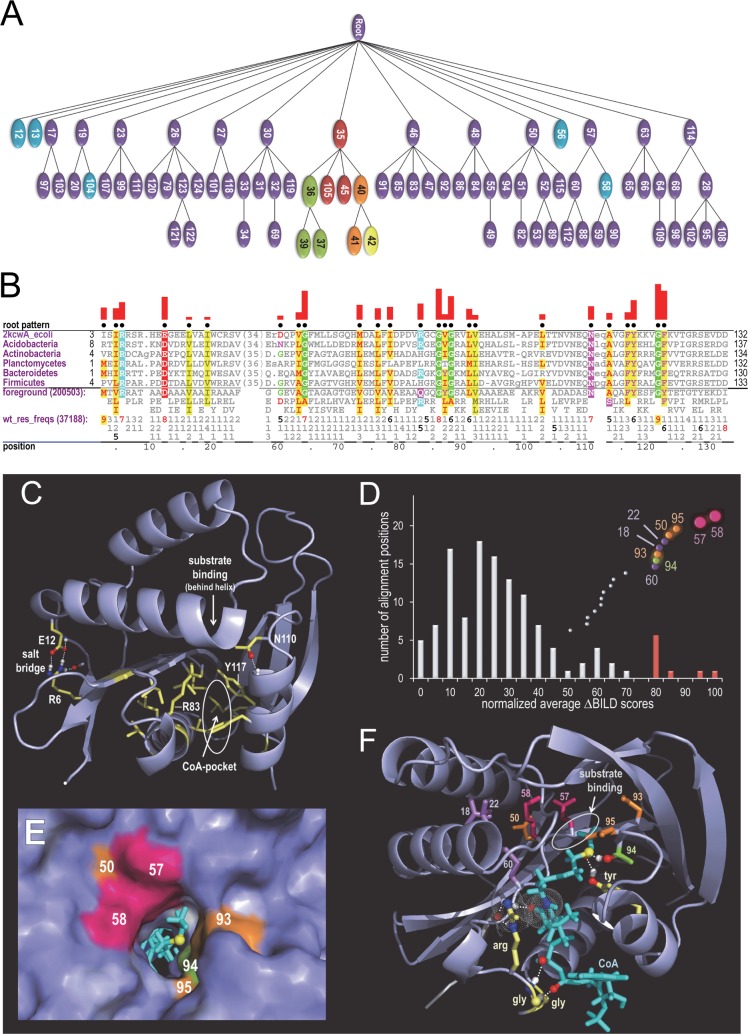
Hierarchy and key features of the acetylase superfamily. **A**. The acetylase hierarchy identified by the sampler. For clarity smaller subtrees not discussed in the text have been omitted; the complete hierarchy is given in **S1.3 Fig**. Purple nodes are not discussed in the text. **B**. Root node “contrast alignment” highlighting conserved patterns most characteristic of acetylases as a whole. Shown are six representative sequences assigned to node-13 of the acetylase hierarchy in (A). These sequences correspond to an uncharacterized prokaryotic acetylase family that conserves all of the root node canonical residues. The sequences are labeled by their bacterial phyla except for the first (proteobacterial) sequence, the structure of which is shown in (C). Below the representative alignment is a summary of the most conserved amino acid residues at each position; the number of sequences (assigned to the foreground) is given in parentheses on the first line. The 1^st^ to 3^rd^ lines show up to three residues at each position that occur both most frequently and in ≥10% of the sequences. Directly below this, the frequencies of the designated residues are given in integer tenths; for example, an ‘8’ indicates that 80–90% of the sequences in the foreground alignment match the corresponding pattern residue. In column 88, for example, glycine occurs in 60–70% and alanine in 20–30% of the sequences. To highlight larger integers ‘5’ and ‘6’ are shown in black and ‘7’-‘9’ in red. The first of these lines (labeled as “wt_res_freqs” for “weighted residue frequencies”) reports the effective number of aligned sequences. In all of these cases, reported frequencies have been down-weighting for redundancy. The black dots above the alignment indicate the pattern positions that were identified by the sampler. Pattern-matching (correlated) residues are highlighted in color, with biochemically similar residues colored similarly. For example, acidic residues are shown in red, basic residue in cyan and hydrophobic residues in yellow; histidine, glycine and proline are each assigned a unique color. The height of the red bars above the alignment quantify (using a semi-logarithmic scale) the degree to which residue frequencies in the foreground diverge at each position from the corresponding positions in the background. In this case, the foreground corresponds to the root node, that is, to the entire tree and thus to all acetylases, and the background corresponds to all proteins unrelated to acetylases, which is represented by standard amino acid residue frequencies. **C**. The acetylase fold with canonical residues most characteristic of the superfamily. The structure show is that of an *E*. *coli* putative N-acetyltransferase assigned to node 13; the corresponding sequence to the first aligned in (B) (pdb_id: 2kcw). **D-F**. Residue positions likely responsible for acetylase functional specificity. **D**. Histogram of normalized average ∆-BILD scores over all column positions. Scores were linearly adjusted so that the lowest score is zero and the highest score is 100. Data points with scores greater than 50 are plotted above the histogram and are spread out vertically to avoid overlap. Histogram bars that are more than two standard deviations above the mean are colored red; corresponding data points are color coded (as explained in text) and enlarged to enhance visibility. Numbers next to data points correspond to the positions of the corresponding aligned columns within the main alignment (i.e., the root node alignment) shown in **S4 Fig.** **E**. Surface representation of the substrate binding pocket showing the locations of six of the residues in (F), which are color coded and numbered as in (D). See text for further details. **F**. Locations within the crystal structure of *Pseudomonas syringae* tabtoxin resistance protein complexed with acyl-CoA (pdb_id: 1gheB)[82] of the nine residues corresponding to the rightmost data points in (D); this protein was assigned to node 104. Residue sidechains are colored as are the data points in (D) and labeled by column positions in the core alignment. In addition, four consensus amino acid residues generally conserved in acetylases are shown in yellow; acyl-CoA is shown in cyan.

N-acetyltransferases constitute a large group of evolutionarily-related proteins that transfer an acetyl ((CH_3_)-(C = O)-) group or, more generally, an acyl (((CH_3_)-(CH_2_)_*x*_-(C = O)-)) group from bound acetyl-Coenzyme A (acetyl-CoA) to a bound substrate. They are implicated in a variety of functions, from bacterial antibiotic resistance to circadian rhythms in mammals. We applied our system to 200,573 protein sequences belonging to the N-acetyltransferases superfamily; this identified a hierarchy consisting of 124 nodes and 83 leaves (**Fig 3A** and **S1.3 Fig**) with most of the subgroups corresponding to hypothetical proteins. For this analysis, we required that at least 200 sequences be assigned to each leaf node in the hierarchy. Due to space limitations, we examine only a few aspects of our analysis; a comprehensive analysis will be published separately. Note that, in the following discussion, we use the more concise term “acetylase” interchangeably with “N-acetyltransferase”.

The residue patterns characteristic of the acetylase superfamily (i.e., across the hierarchy) are highlighted in **Fig 3B**. Except for an isolated salt bridge involving Arg6 and Glu12, these residues cluster within the coenzyme A (CoA)-binding region (**Fig 3C**) and correspond to the following acetylase structural features: (i) An arginine/glutamine (Arg 83 in **Fig 3C**) that forms a hydrogen bond with a backbone oxygen and that contacts bound CoA, which is required for the acetyltransferase reaction. This contact appears to involve a π-orbital stacking interaction between a CoA–CO-NH- group and either the guanidinium group of arginine or the–CO-NH2 group of glutamine. (ii) Two glycine residues (Gly86 and Gly88 in **Fig 3B**), the backbone atoms of which form hydrogen bonds with bound CoA. (iii) A tyrosine residue (Tyr117 in **Fig 3B and 3C**), which, for some acetylases, has been proposed to function as a general acid [82, 83] facilitating transfer of the acetyl group from the sulfur atom of CoA to the substrate. (iv) A nearby asparagine residue (Asn110) that may assist in this process. (v) Two other (typically aromatic) pattern residues (Phe116 and Phe122) that pack against the tyrosine, one of which may form π-π stacking interactions with the (aromatic) adenine group of CoA. (vi) The remaining canonical residues form hydrophobic contacts that presumably facilitate the core structure of this superfamily.

### Most discriminating aligned column positions

In addition to the above mentioned known properties, our analysis reveals new information about underlying properties of acetylases. We identified, for example, those residue positions most likely responsible for functional specificity, as follows: For each major subgroup (i.e., each subtree directly attached to the root) at each column position, we computed the sum of two BILD scores: one score for that subgroup alone and another for the entire superfamily absent that subgroup. BILD scores quantify the information content within aligned columns based on the (weighted) amino acid residue counts. Next, we computed a Δ-BILD score, defined as the difference between this sum and the BILD score for the superfamily as a whole. Taking the difference between two BILD scores in this manner may be understood as constructing a BILD score from two alternative hypotheses of relatedness among the residues in a column [78]. In this context, a column’s Δ-BILD score will be positive when the sequences assigned to a major subgroup and those assigned to the rest of the tree have distinct residue compositions. Hence, each Δ-BILD score quantifies the degree to which a given subgroup amino acid composition diverges at a given column position from the composition of the superfamily as a whole. Finally, we compute the average Δ-BILD score for each column position. The histogram in **Fig 3D** plots these average scores over all columns within the acetylase core alignment. Those columns with the most extreme average Δ-BILD scores correspond to aligned residue positions that are most highly conserved within each major subgroup but that are poorly conserved in the superfamily as a whole—suggesting that residues at high-scoring positions are important for subgroup functional specialization.

Nine column positions stand out in this way (**Fig 3D**). As is illustrated in **Fig 3E and 3F**, which highlights the structural locations of these residues in a protein assigned to node-104 of our hierarchy (**Fig 3A**), residues in six of these positions line the substrate binding and catalytic sites. This is as expected, since residues in this region are likely to have a major influence on substrate and catalytic specificities. The two residues with the most extreme scores (which are colored red in **Fig 3E and 3F** and which correspond to columns 57 and 58) are directly adjacent to the substrate binding site. Structurally behind these two residues is another high scoring position (column 50 of the core alignment), the residue at which may be important for maintaining the sidechains of these other residues in a functionally-optimal conformation. Likewise, the residue at position 60 interacts both with the position 58 residue and with bound CoA, both of which may be important for orienting the substrate and CoA for enhanced catalytic efficiency and specificity. Three other high Δ-BILD positions (columns 93–95) likewise line the substrate binding pocket (**Fig 3E and 3F**). In particular, the column-94 threonine residue in **Fig 3F** forms a hydrogen bond with the CoA sulfur atom and thus may play a role in catalysis. The two remaining high Δ-BILD scoring positions (columns 18 and 22) neither line the substrate binding pocket nor interact with CoA, but may perform a currently unknown functional role (e.g., see subtree 35 analysis below).

### Structural partitioning into CoA- and substrate-associated subdomains

**Fig 4:**
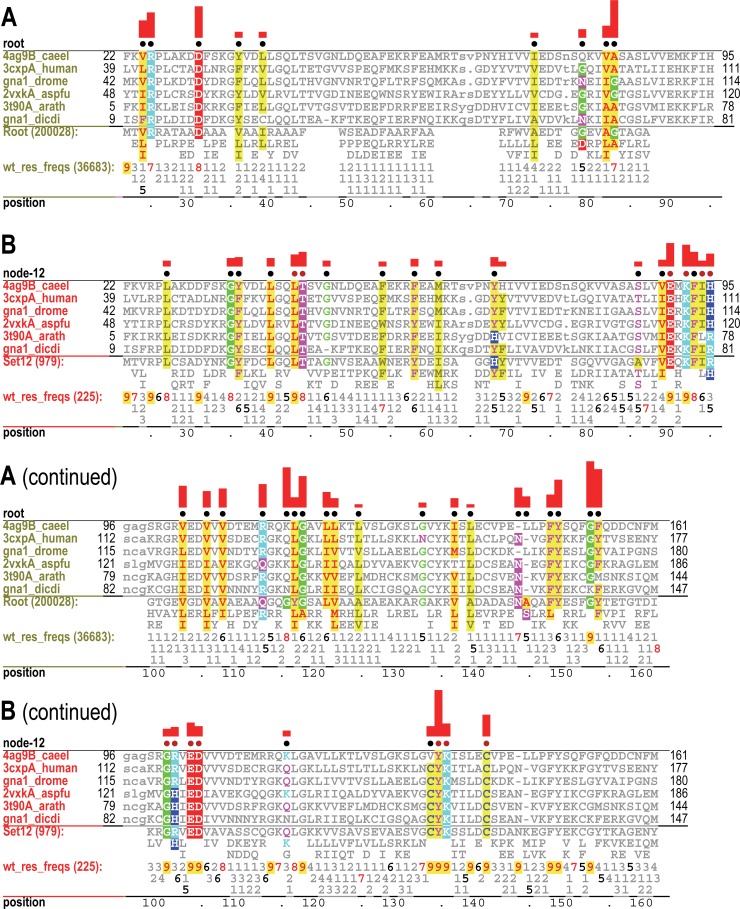
Correlated residue patterns associated with the node 12 lineage. See the legend to **Fig 3B** for an explanation of notation. The same representative node-12-assigned sequences are shown in both A and B, but highlight pattern residues most distinctive of the root (i.e., the superfamily) and of the node 12 subgroup, respectively. **A**. Contrast alignment corresponding to the root of the acetylase hierarchy. **B**. Contrast alignment corresponding to node 12 of the hierarchy. Here the foreground corresponds to sequences assigned to node-12 and the background to all other acetylase sequences. The highlighted columns below the red dots correspond to the residues shown in **Fig 5B**; note that the constraints imposed on these residues (i.e., the heights of the red bars above the dots) are generally higher than the constraints imposed on the other pattern residues.

**Fig 5:**
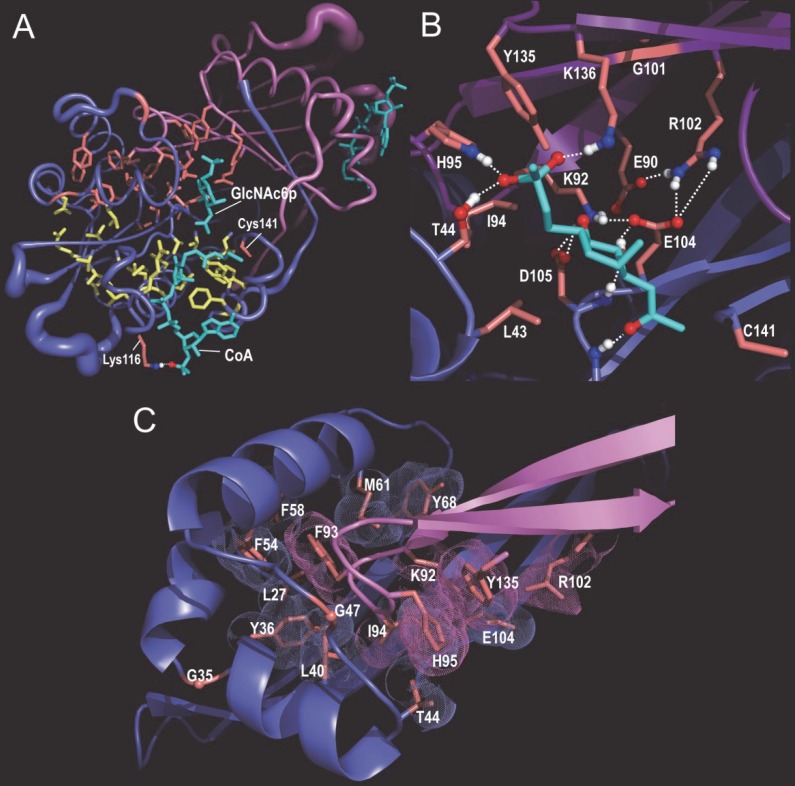
The *Caenorhabditis elegans* glucosamine-6-phosphate N-acetyltransferase (Gna1) complexed with CoA and N-acetylglucosamine-6-phosphate (GlcNAc6p) (pdb_id: 4ag9) [84]. Gna1 was assigned to node 12 of the hierarchy. **A**. Structural locations of acetylase residues (yellow) and node 12-specific residues (red). **B**. Node 12-specific residues involved in substrate binding. These residue positions are indicated in **Fig 4**(as red dots above column positions). **C**. Node 12-specific residues associated with the homodimeric interface.

Just as residues characteristic of the acetylase superfamily as a whole cluster around the CoA-binding pocket (**Fig 3B and 3C**), major-subgroup-conserved residues tend to cluster together in another, distinct region of the domain, one that again appears to be associated with substrate and catalytic specificity. To illustrate this, the analysis in **Fig 4A and 4B** show both categories of correlated residues for representative sequences assigned to node-12 of the acetylase hierarchy. **Fig 5A** shows how these two types of residues structurally cluster into two halves of the acetylase domain [84]. Two node-12 residues, however, are outside of the main node 12 cluster; these correspond to Lys116 and Cys141 of *Caenorhabditis elegans* glucosamine-6-phosphate N-acetyltransferase (Gna1) (pdb_id: 4ag9). Lys116, which replaces the glycine residue typically found at this position in the acetylase superfamily, interacts with a phosphate group of CoA, thereby possibly helping position CoA for catalysis. Cys141 is an active site residue that covalently links to the sulfur atom of CoA [84]. Essentially all of the remaining node-12 residues are involved either in substrate binding or homodimer formation or both. As do other acetylase subgroups, this subgroup forms a homodimeric complex [84]. Node-12-conserved residues from both subunits together bind the substrate (**Fig 5B**). Some of these residues and nearly all of the remaining node-12 residues form the homodimeric interface (**Fig 5C**). Together these observations rationalize the conservation of node-12 residues and suggest that they form a structural and biochemical environment favoring specific acetylation of glucosamine-6-phosphate. The high Δ-BILD scores for columns 18 and 22 (**Fig 3D and 3F**) (corresponding to Leu40 and Thr44 in Fig 5C, respectively) might be rationalized, at least in this case, to their role in homodimerization; an additional rationalization for column 22 is binding to substrate, as this is seen for Thr44 in Fig 5B.

### The Gna1 analysis compared with that of other methods

As just described, our analysis of the Gna1 subgroup identifies two categories of residues that are structurally partitioned (in a strikingly non-random manner) into two subdomains (**Fig 5A**). Moreover, functional roles for these pattern residues in dimerization, in substrate- and CoA-binding and in catalysis are easily rationalized based on their structural locations. Because no structural information was provided to our procedures (steps 1–3 in **Fig 2A**), this indicates that the identified correlated residue patterns correspond to true biochemical properties important for protein function. Given these results, we investigated whether functional residue prediction programs specifically designed to identify catalytic, ligand-binding and subtype-specific residues yield similar results. **S2.1A–S2.1D Fig** compares our analysis of the Gna1 subgroup to that of two such programs: FRpred [43] and CLIPS-1D [45]. This reveals that the structural bipartitioning of residues is unique to our hiMSA analysis, which therefore, at least in this case, is finding protein structural features that these other methods fail to identify.

### Gna1 compared with other acetylases

The structural bi-partitioning phenomenon characteristic of the Gna1 subgroup is also observed within other subgroups of the acetylase superfamily. This is observed, for example, for a subgroup (node 56 in **Fig 3A**) that includes Hpa2 histone acetyltransferases (pdb_id: 1qsm; **S2.1E Fig** and **S2.2 Fig**) and for a subgroup (node 58 in **Fig 3A**) that includes the amino-terminal acetyltransferase catalytic subunit Naa10 (pdb_id: 4kvo)(**S2.1F Fig** and **S2.3 Fig**). Hence, many acetylases appear to be composed of two components: a CoA-binding subdomain and, positioned next to this, a substrate binding and reaction chamber. Our analysis of the Naa10 subgroup also reveals a third category of pattern residues corresponding to a third structural partition that forms a surface layer over the two other partitions and that might mediate binding to other proteins.

### hiMSA analysis suggests an acetylase induced-fit mechanism

To further decompose the sequence determinants of protein function, hiMSA analysis expands major subgroup nodes into subtrees. Node-35 in **Fig 3A**, for example, has been expanded into a subtree consisting of 9 nodes. Contrast hierarchical alignments of sequences in the lineage from the root node to node-42 and from the root node to node-39 are shown in **S3 Fig**. We may interpret these alignments probabilistically in terms of the underlying hiHMM distribution, as follows: The root pattern residues (highlighted in the upper alignment of **S3A Fig**) correspond to those sequence properties defining the main probability cloud of the sequence distribution for the acetylase superfamily. Likewise, the node-35 pattern residues (highlighted in the lower alignment of **S3A Fig**) define a probability subcloud within this main cloud. Note that, although the aligned sequences in **S3A Fig** are assigned to distinct nodes (either node-42 or node-39) within the node-35 subtree, both categories of sequences conserve well the root and node-35 pattern residues. This presumably corresponds to their shared functional constraints. In contrast, the node-40 pattern residues highlighted in **S3B Fig** are distinct from the node-36 pattern residues highlighted in **S3C Fig**. We hypothesize that this reflects these subgroups’ functional divergence and corresponds to distinct subclouds within the node-35 cloud. Likewise the node-42 and node-39 pattern residues define sub-subclouds within these subclouds.

**Fig 6:**
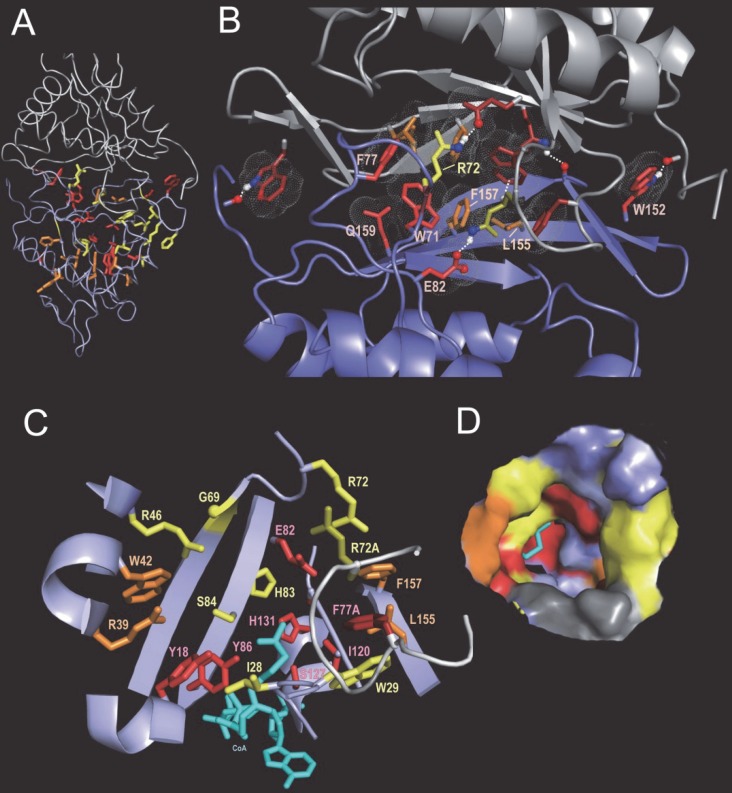
Pattern residues associated with the node 42 lineage. **A**. The structural locations of pattern residues corresponding to nodes 35, 40 and 42 (the sidechains of which are shown in red, orange and yellow, respectively) of the apo form of a putative acetylase from *Salmonella typhimurium* (pdb_id: 3dr6). This protein forms a homodimer, the two subunits of which are shown in blue and gray. Residues are shown within the bottom subunit of the homodimeric complex only. **B**. Pattern residues associated with interactions between dimeric subunits in the apo form (pdb_id: 3dr6). **C**. Pattern residues that line the substrate binding pocket within the CoA-bound form of the same acetylase as in (A) (pdb_id: 3dr8). CoA is shown in cyan. **D**. The corresponding surface plot of the pocket using the same color scheme.

**Fig 7:**
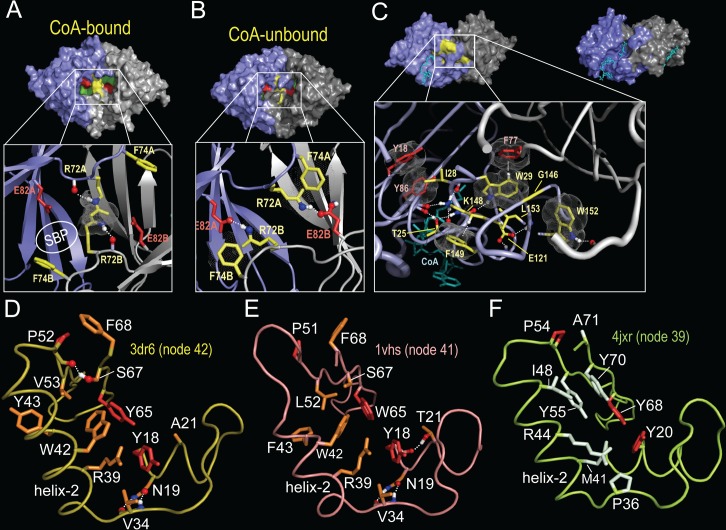
Node 35 structural features implicated in a proposed induced-fit mechanism. **A-C**. Conformational changes that involve two conserved residues and that mediate opening and closing of the substrate binding pocket of a putative acetylase from *Salmonella typhimurium* with and without bound acetyl-CoA (pdb_id: 3dr8 and 3dr6). **A**. (top) Surface view of the open conformation when CoA is bound to both subunits (pdb_id: 3dr8). The surface of Glu82 is shown in red, of Arg72 in yellow and of the rest of the substrate binding pocket (SBP) in green. (bottom) Close up view of Glu82 and Arg72 at the dimeric interface (pdb_id: 3dr8); the SBP is indicated. **B**. (top) Surface view of the closed conformation when neither subunit is bound to CoA (pdb_id: 3dr6). Note that the substrate binding pocket appears inaccessible. (bottom) Close up of the Glu82-Arg72 salt bridge formed at the subunit interface. **C**. (*top left*) Surface side view of the acetyl-CoA bound form (pdb_id: 3dr8) showing the locations of a cluster of Set42-specific residues (shaded yellow). CoA is shown in cyan. (*bottom*) The same view of 3dr8 as in A but rotated by 90 degrees to show a side view. The expanded box shows the node-42 pattern residue interactions forming a bridge between adjacent loop regions. (*top right*) A similar view of *C*. *Elegans* Gna1 (pdb_id: 4ag9) showing the adjacent locations of the CoA and substrate within a channel rather than a pocket as in (A). **D-F**. Differences between node 40 and node 36 pattern residues. Residues with red sidechains correspond to node 35 pattern residues. See **S3 Fig** for the contrast alignment showing pattern residues. **D**. Node 40 pattern residues (orange sidechains) within 3dr6 (an acetylase assigned to node 42). **E**. Node 40 pattern residues (orange sidechains) within 1vhs (an acetylase assigned to node 41). **F**. Node 36 pattern residues (light green sidechains) within 4jxr (an acetylase assigned to node 39).

**Fig 8:**
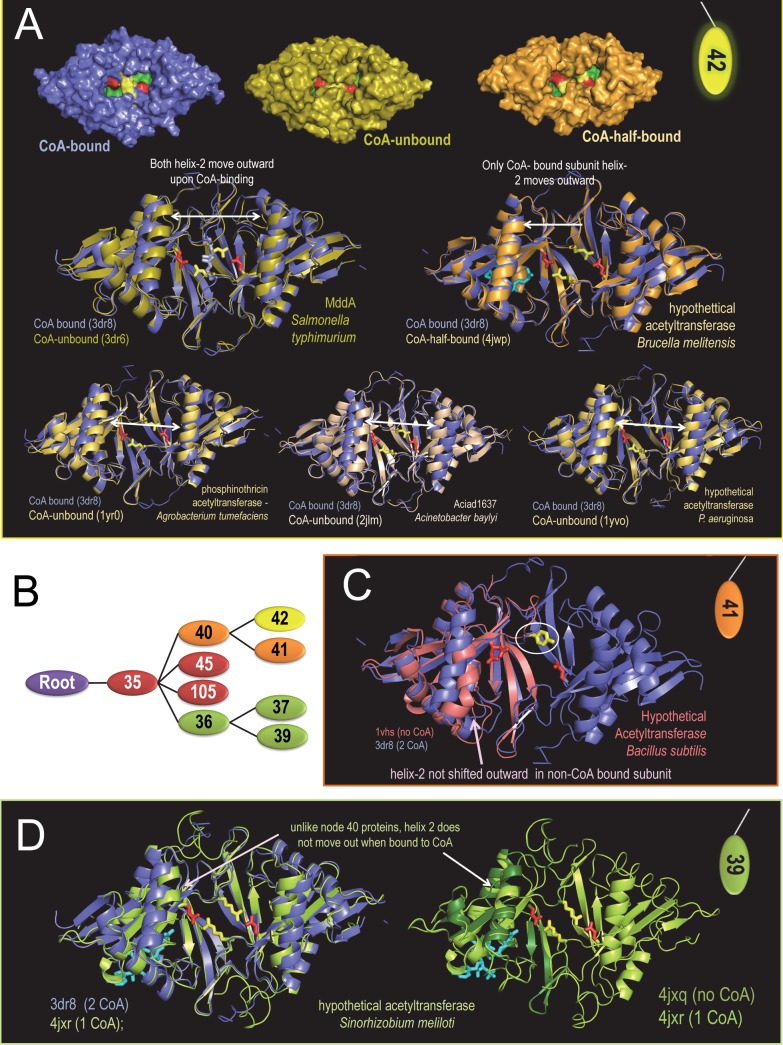
Opening and closing of the substrate binding pocket and movement of helix 2 based on superpositioning of bound and unbound acetylase domains. Sidechains of the putative induced fit glutamate and arginine residues in the closed form are shown as red and yellow sticks, respectively. CoA within half-bound homodimers is shown as cyan colored sticks. **A**. Node 42 CoA-bound, CoA-unbound and CoA-half-bound structures. When bound to CoA, helix-2 moves outward relative to the unbound homodimer. The sidechain of the 3dr8 arginine residues (open conformation) for the 3dr8 vs 3dr6 superposition is shown as light blue sticks (compare with Fig 7A). **B**. Node 35 hierarchy with color coding. **C**. Node 41 unbound monomeric structure superimposed over the node 42 CoA-bound homodimer. Note that the proposed induced-fit arginine residue is replaced by a tyrosine, which could also form both a hydrogen bond to the glutamate residue and a π- π stacking interaction with this tyrosine from the other homodimeric subunit. **D**. Node 39 superpositions showing that, unlike sequences assigned to the node 40 subtree, helix 2 does not appear to move outward upon binding to CoA. This may be due to differences between node 40 and node 36 pattern residues associated with this helix (see Fig 7D–7F).

Examining these node-specific pattern residues within proteins of known structure suggests both shared and divergent functional and structural roles. For example, **Figs 6–8**show the locations of pattern residues characterizing the lineage from node-35 to node-42 within CoA-bound and unbound structures. These structures are of an acetylase from *Salmonella typhimurium* that forms a homodimeric complex (pdb_ids: 3dr8 and 3dr6, respectively, determined in a Structural Genomics unpublished study) and that corresponds to the *Salmonella enterica* MddA (methionine derivative detoxifier) protein [85]. **Fig 6A** shows, within one dimeric subunit, the locations of the most discriminating pattern residues associated with nodes 35, 40 and 42. **Fig 6B** reveals that about half of the node-35 pattern residues (red sidechains) interact with the adjacent dimeric subunit, suggesting a role for these residues in dimer formation. This dimeric interface is strikingly different from the node-12 dimeric interface shown in **Fig 5C**—suggesting that subgroup-specific residues have evolved to accommodate different dimeric (and possibly higher-level multimeric) complexes. Two node-40 and one node-42 pattern residues also occur at this interface, about which more will be said below. The substrate pocket is formed by four of the node-40 pattern residues as well as by seven node-35 and seven node-42 pattern residues (**Fig 6C and 6D**), which together presumably contribute to substrate specificity. We conjecture that those residues associated with higher- and lower-level nodes (e.g., node-35 and node-42, respectively) recognize similar and distinct substrate features, respectively. The substrates for these proteins are currently unknown.

A comparison between CoA-bound and unbound forms of MddA suggests a role for the second most striking node-42 pattern residue in **S3 Fig**, namely Arg72. In the CoA-bound state (**Fig 7A**): (i) Arg72 forms a guanidinium-group π-π stacking interaction [86–88] with the same arginine from the other subunit; (ii) Glu82, a Node-35 pattern residue that corresponds to the column 57 residue in **Fig 3D** and thus may play a key role in substrate binding and/or catalysis, is free to bind to substrate; and (iii) Phe74, another Node-42 residue, is in an open, substrate-accessible configuration. However, in the unbound state (**Fig 7B**): (i) Arg72 forms a salt bridge with Glu82; and (ii) Phe74 undergoes a conformational change that blocks access to the substrate-binding pocket. The Arg-Glu salt-bridge interaction is also seen in unbound structures of related acetylases conserving this arginine residue (see below). Note that in this state the salt bridge blocks substrate access to Leu155 and Phe157, which line the substrate binding pocket. Together these changes appear to close the pocket, as surface views of the CoA-bound and unbound structures reveal (**Fig 7A and 7B**).

Note, however, that a substrate of MddA, namely L-methionine sulfoximine (MSX), binds to a related acetylase from *Pseudomonas aeruginosa* (PA4866; pdb_id: 2j8r) [89] in the absence of bound CoA and with the corresponding Arg-Glu salt bridge intact. Hence, in the absence of CoA the substrate binding pocket is sufficiently accessible to allow MSX binding in this case. Likewise, in another non-CoA-bound structure of the same protein, glycerol (which was used as a cryoprotectant) (pdb_id: 2j8m) [89] also binds to the substrate pocket.

Some acetylases form a CoA- and substrate-binding channel instead of a distinct substrate binding pocket (compare surface views of MddA and *C*. *Elegans* Gna1 in **Fig 7C**). It is perhaps for this reason that nine Node-42 pattern residues form a bridge across this channel (box in **Fig 7C**), thereby forming a donut-hole between the CoA and substrate binding sites and a closeable pocket essential for the proposed CoA-induced-fit substrate-binding mechanism. Other, non-node-42 subgroups of node-35 also form such a bridge, albeit utilizing different pattern residues and interactions suggesting functional divergence and specialization.

### Possible roles for helix-2 and Node 40 residues in an induced-fit mechanism

Ten of the Node-40 pattern residues are associated with helix-2 of the acetylase domain (**Fig 7D–7E**). This raises questions regarding the role of these residues and of helix-2. Upon binding to two CoA molecules, disruption of the Arg-Glu salt bridge, and opening of the substrate pocket, helix-2 of each dimeric subunit moves outward relative to helix-2 of the other subunit. This is seen in structural superposition of CoA-bound and unbound forms of Gna1 (**Fig 8A**). Corroborating this notion, the helix-2 pairs are also closer together within homodimeric structures of various unbound forms of Node-42 acetylases (**Fig 8A**). And when only one dimeric subunit is bound to CoA, helix-2 in that subunit moves outward, but not helix-2 in the other, unbound subunit (**Fig 8A**). In this half-bound state the pocket appears to open partially, but the Arg-Glu salt bridge remains intact. Thus the proposed induced-fit mechanism appears to require CoA binding to both subunits to allow substrate access to the binding site.

If movement of helix-2 is mediated by the ten Node-40 residues associated with this helix (at least in part), then we might expect acetylases assigned to both of its child nodes, namely nodes 41 and 42 (**Fig 8B**), to share this mechanism, but not other Node-35 acetylases. Consistent with this notion, for an unbound form of an acetylase assigned to Node-41, helix-2 exhibits an inward-shifted conformation (**Fig 8C**), as do unbound Node-42 acetylases (**Fig 8A**). In this Node-41 acetylase the induced-fit arginine residue is replaced by a tyrosine, which, nevertheless, could still form a hydrogen bond to the Glu residue in a closed conformation, and π-π stacking interactions with the corresponding tyrosine from the second dimeric subunit in an open conformation. Solving the crystal structures of CoA-bound forms of this and other Node-41 acetylases will test this hypothesis. In contrast, helix-2 of an acetylase assigned to Node 39 is associated with distinct pattern residues (**Fig 7F**). Upon binding to one CoA molecule, helix-2 of this acetylase fails to move outward (**Fig 8D**), which is not the case for the Node-42 CoA-half-bound acetylase mentioned above (**Fig 8A**). Taken together, these observations suggest that conformational changes in helix-2 mediated by Node-40 pattern residues may play a role in transitioning between the open and closed states. In particular, the two occurrences of this helix move further apart in the open, CoA-bound state thereby facilitating access to the substrate binding pocket. The node-40 pattern residues associated with helix-2 may ensure proper positioning of this helix upon transitioning into the open state.

## Discussion

As illustrated here, a hiMSA analysis can help make sense of uncharacterized or poorly characterized protein sequences and structures. Identifying correlated features of the empirical sequence and structural properties in this way provides a more objective basis for genome annotation than attempting to predict unobserved biochemical properties. We have illustrated here how a hiMSA analysis can provide new biological insights into characterized proteins and how it complements other methods for characterizing pairwise correlated residues [8, 90–93] by finding correlated residue patterns presumably associated with functional specialization. hiMSA analysis advances sequence-based analysis on three fronts, as follows:

**(1)** It models both site-specific and correlated features of an entire major protein superfamily concurrently and in a statistically coherent manner. This is important because an ideal model should explain all relevant features of the input sequences; this, in turn, requires searching the space of possible models using, as the objective function, each model’s likelihood of having generated the input data. Rather than relying on separate programs to pre-compute a tree, an alignment, or domain profiles, it optimizes modeled features based on consistent, statistically-based criteria. One reason that ad hoc approaches are often used is that the likelihood distribution over possible models is highly complex: Proteins typically have evolved into hierarchically-arranged, functionally-divergent subgroups, each of which exhibits both properties unique to that subgroup and others shared with related subgroups or with the superfamily as a whole. Hence, optimization over a complex distribution using deterministic methods rapidly becomes intractable as the number of sequences increases. A hiMSA analysis overcomes these barriers by using MCMC sampling [20], which has widely proved to be the most effective approach for exploring a complex, high-dimensional and highly-correlated probability distribution. MCMC sampling accomplishes this by sampling iteratively according to the conditional distribution of each variable in turn.

**(2)** It avoids modeling extraneous features and random noise. This is important because over-parameterized models are computationally inefficient and their parameters over-fitted and, of course, modeling noise yields spurious results. To address this, here we applied the MDL principle [65], which provides a criterion for choosing among alternative models for describing a set of data. This ensures, for example, that sequence regions are aligned and correlated patterns defined *only* when justified statistically. Likewise, for a major protein superfamily, which typically consists of more than 25,000 sequences, a phylogenetic tree is far too detailed and uncertain to be reliable. Instead, our analysis models each superfamily more simply, as hierarchically-arranged sequence subgroups, each of which is defined by the pattern that most distinguishes it from closely-related subgroups. At the same time, it adjusts posterior probabilities to account for differences in complexity between competing model architectures. This ensures creation of a hierarchical alignment only when justified statistically.

**(3)** It allows the sequences themselves to reveal their statistically most striking features rather than attempting to identify predetermined types of residues. This is important because, due to the incompleteness of protein experimental annotations, training a program to recognize a predetermined subset of residues—namely those with characterized (and therefore annotated) roles, such as in catalysis and substrate recognition—biases it against correlated residue patterns with important but uncharacterized roles. Although programs trained in this way are useful, there is a need as well for the alternative approach described here. At the same time, hiMSA analysis may probabilistically predict protein function through co-classification with proteins of known function. In this way, it seeks to discover, through statistical inference, biological phenomena that thus far have evaded detection—as did classical genetic analyses.

In summary, hiMSA analysis comprehensively models an entire major protein superfamily in a statistically coherent manner while avoiding both under- and over-parameterization. This allows the sequences themselves to reveal biologically-relevant features, leading to the generation of plausible hypotheses for experimental follow up, as illustrated by the acetylase analysis described here.

## Methods

### Background

This section reviews our previous MSA and BPPS statistical models used for hiMSA analysis.

*Notation and Definitions*. The following notation is used for vectors **v** = (*v*_1_,…,*v*_*n*_)^*T*^ and **w** = (*w*_1_,…,*w*_*n*_)^*T*^: |**v**| = |*v*_1_|+…+|*v*_*n*_|, **v** + **w** = (*v*_1_ + *w*_1_,…,*v*_*n*_ + *w*_*n*_)^*T*^, **v**/**w** = (*v*_1_/*w*_1_,…,*v*_*n*_/*w*_*n*_)^*T*^, $v^{w}=v_{1}{}^{w_{1}}\ldots v_{n}{}^{w_{n}}$, and Γ(**v**) = Γ(*v*_1_)…Γ(*v*_*n*_). Given *K* proteins, their sequences are defined by $R={\left(R_{1}^{T},\ldots ,R_{K}^{T}\right)}^{T}$ where each vector $R_{k}=\left(r_{k,1},\ldots ,r_{k,n_{k}}\right)$ corresponds to the *k*-th sequence, *n*_*k*_ is the *k*-th sequence’s length and the *r*_*k*,*i*_ corresponds to the *i*-th residue in that sequence. **h**( ) defines a counting function where, for example, **h**(*R*_*k*_) returns a length 20 vector of the counts for the residue types in *R*_*k*_; 〈.,.〉 denotes the inner product of 2 vectors.

*MSA sampling*. Given a sequence alignment of *w* columns, the set of variables defining the sequence positions for column *j* is defined by *A*_*j*_ = {*a*_1,*j*_,…,*a*_*K*,*j*_} so that the alignment is defined by the matrix **A** = (*A*_1_,…,*A*_*w*_)^*T*^. We define *A*_*j*[−*k*]_ ≡ *A*_*j*_ − {*a*_*k*,*j*_} to denote the set *A*_*j*_ without *a*_*k*,*j*_ and define {**A**} ≡ {*a*_*k*,*j*_: *k* = 1,..,*K*, *j* = 1,…,*w*} to denote the set of residues indices for the alignment variable **A**. We represent the collection of residues indexed by elements in a set C as **R**_*C*_. For instance, **R**_{**A**}_ = {*a*_*k*,*j*_: *k* = 1,…,*K*; *j* = 1,…*w*} represents the set of residues in the alignment defined by **A**.

Ignoring insertions and deletions (indels), the MSA posterior distribution [69–71] is defined by:

$$\pi \left(R|X\right)\propto \Gamma \left(h\left(R_{{\left\{X\right\}}^{c}}\right)+\beta_{0}\right)\cdot \prod_{j=1}^{w}\Gamma \left\{h\left(R_{\left\{X_{j}\right\}}\right)+\beta_{j}\right\}$$ (1)

where **X** defines a *w* column alignment; **R** specifies the sequences; $R_{\left\{X_{j}\right\}}$ and $R_{{\left\{X\right\}}^{c}}$ specify the residues in column *j* of the alignment and outside of aligned columns, respectively; and **β**_*j*_ and **β**_0_ specify the numbers of pseudocounts in each column *j* and in the background, respectively. We model indels based on the **hidden Markov model (HMM)** shown in **S1.1 Fig**. The sampler infers position-specific gap penalties, as follows: For a given alignment, each sequence is associated with a “path” through the HMM indicating its alignment against the model. We denote the collection of these paths by Λ, the total number of HMM transitions of type M→M, M→I, …, D→D at position *j* by *N*_*mm*_[*j*], *N*_*mi*_[*j*], …, *N*_*dd*_[*j*], and corresponding prior pseudocounts by *n*_*mm*_,…,*n*_*dd*_. The contribution of implicit gap penalties to the overall probability, after integrating out position-specific transition probabilities ($\vec{\iota },\vec{\delta }$), is:

$$\begin{array}{c} \pi (\Lambda )=\iint \pi (\Lambda |\vec{\iota },\vec{\delta })P(\vec{\iota },\vec{\delta })d\vec{\iota }d\vec{\delta }=\prod_{j=1}^{w}[\frac{\Gamma (N_{mi}\left[j\right]+n_{mi})\Gamma (N_{md}\left[j\right]+n_{md})\Gamma (N_{mm}\left[j\right]+n_{mm})\Gamma (n_{m\cdot })}{\Gamma (N_{m\cdot }\left[j\right]+n_{m\cdot })\Gamma (n_{mi})\Gamma (n_{md})\Gamma (n_{mm})}\\ \times \frac{\Gamma (N_{ii}\left[j\right]+n_{ii})\Gamma (N_{im}\left[j\right]+n_{im})\Gamma (n_{i\cdot })}{\Gamma (N_{im}\left[j\right]+N_{ii}\left[j\right]+n_{i\cdot })\Gamma (n_{ii})\Gamma (n_{im})}\times \frac{\Gamma (N_{dd}\left[j\right]+n_{dd})\Gamma (N_{dm}\left[j\right]+n_{dm})\Gamma (n_{d\cdot })}{\Gamma (N_{dd}\left[j\right]+N_{dm}\left[j\right]+n_{d\cdot })\Gamma (n_{dd})\Gamma (n_{dm})}]. \end{array}$$ (2)

This gives rise to a new posterior distribution

P(X,Λ)∝π(R|X,Λ)×π(Λ), (3)

for which the transition probability parameters need not be fixed or updated and which allows the gap penalties to be determined from the sequence data [63, 71].

*BPPS sampling*. A hierarchical alignment consists of a MSA, a tree—each node of which corresponds to a **contrast alignment** (**Fig 2B**)—and a set of labels assigning each aligned sequence to one of the nodes. Each contrast alignment consists of a pattern and a **foreground** or a **background** partition corresponding, respectively, to the subtree rooted at that node and to the rest of the subtree rooted at the parent of that node. By sampling over alternative sequence and pattern assignments, our BPPS sampler seeks to optimally partition the sequences into subgroups where the foreground sequences conserve a pattern that the background sequences do not [21]. MCMC sampling is required because, *a priori*, we know neither which sequences belong to each node, nor which positions are pattern positions, nor which residues are conserved at each pattern position. The sampler also seeks to optimize the number of nodes and the relationships between nodes (**Fig 2C**). Thus the sampler favors convergence on a hierarchy where the pattern and partitioning for each node best distinguishes its foreground from its background. The probability of the data given the parameters of the BPPS statistical model is defined (logarithmically) as:

$$\log\;P\left(X|H,S,A,\alpha ,\Theta \right)=\sum_{h=1}^{\left|H\right|}\left(\sum_{z\in H_{h}^{+}\cup H_{h}^{-}}\sum_{i\in S_{z}}\sum_{j=1}^{k}\left\langle \log\;\theta_{h,j},x_{ij}\right\rangle +\sum_{z\in H_{h}^{+}}\sum_{i\in S_{z}}\sum_{j=1}^{k}I_{A_{h,j}}\left\langle \log\frac{\theta_{h,j}^{\alpha_{h}}}{\theta_{h,j}},x_{ij}\right\rangle \right)$$ (4)

where **X** defines a sequence alignment of *k* columns, the probability of which is defined by **Eq (3)** and from which the BPPS sampler infers **H**,**S**,**A**,**α**, and **Θ**; **H** defines the hierarchy with $H_{h}^{+}$ and $H_{h}^{-}$ denoting subgroup *h* foreground and background node sets, respectively; **S** specifies assigned sequence sets for each node; **A** defines foreground pattern residue sets where $A_{h,j}=\emptyset \to I_{A_{h,j}}=0$ else $I_{A_{h,j}}=1$; **α** specifies the fraction of background ‘contamination’ at pattern positions in the foreground; **Θ** specifies residue compositions with $\theta_{h,j}^{\alpha_{h}}\equiv \left(1-\alpha_{h}\right)\theta_{h,j}+\alpha_{h}A_{h,j}$ specifying the foreground and **θ**_*h*,*j*_ the background. See [21–24] for details.

### The hiHMM/hiMSA sampler

Steps 1 and 2 in **Fig 2A** are accomplished using our recently described MSA [63] and BPPS [23, 24] samplers, respectively. This results in a *w*-column alignment, the sequences of which are partitioned into hierarchically-arranged subgroups. Converting this into a hiMSA (Step 3 in **Fig 2A**) involves the following: **(i)** Applying MSA sampling to refine the alignment of each set of subgroup sequences, starting with the root node’s child subtrees and recursively progressing to descendant subtrees. This will extend sub-alignments into subgroup-specific insert regions and will remove regions from each sub-alignment that are deleted relative to its corresponding super-alignment (see **S1.2 Fig**). To improve speed, all but one among each set of highly similar sequences (e.g., ≥ 95% identity) may be removed. **(ii)** The lineage-specific alignments are aligned to each other (based on the hierarchy) to construct a set of template alignments, as illustrated schematically in **S1.2C Fig**. These templates define how each of the subtrees is aligned to its parent node, which is important for defining BPPS foreground and background frequencies (see Eq (4)). **(iii)** Apply BPPS sampling to find additional pattern positions within subgroup-specific insert regions. Because these insert regions are absent from the background alignment, standard amino acid residue frequencies are used as the background. **(iv)** Finally, BPPS sampling is performed to further refine the hiMSA and thus the hiHMM.

This process creates a hierarchical alignment, for which the sequences assigned to each subtree in the hierarchy are more or less optimally aligned for that subtree. In other words, only those regions that are conserved across each subtree’s sequence set are aligned—as is illustrated schematically in **Fig 2D**. The hiHMM defined by this process can be used to search the protein database using MAPGAPS [67].

### Visualizing sequence information

Since the focus of our analysis is on large, functionally diverse protein superfamilies, hiHMM sampling produces a large amount of output. To make this output intelligible, several procedures are used to summarize the results. First, for each leaf node hierarchical contrast alignments (as shown schematically in **Fig 2B**) are created. This is illustrated in **S2.2, S2.3** and **S3 Figs**, which show the set of contrast alignments corresponding to the lineages from the root to various leaf nodes, with one contrast alignment for each node along the lineage. In essence, each lineage-contrast-alignment decomposes the constraints imposed on the sequences assigned to that leaf node. This is perhaps the most important information obtained from the analysis. These lineages, one for each of the leaf nodes, are concatenated into a single rich text format (rtf) file as output. Another previously-described [76] procedure evaluates the run-to-run consistency of the hierarchical alignment obtained.

### Implementation

The key steps of hiMSA analysis (**Fig 2A**) were implemented in the following C++ programs: MAPGAPS [67] identifies and initially aligns related sequences and the GISMO sampler [63] improves the alignment (step 1). The omcBPPS sampler [23, 24] classifies the aligned sequences into a hierarchy (step 2). The hieraln and hierview programs (first described here) create and optimize a hiMSA and output lineage-specific alignments, respectively (step 3). MAPGAPS can use an existing hiMSA to identify, align and hierarchically classify previously identified and new database sequences. Finally, the hierview program also creates output files for viewing key structural features using PyMOL (step 4), as was illustrated here.

## Supporting Information

S1 Fig The hiHMM, hiMSA and full acetylase hierarchy. (PDF)

S2 Fig Gna1 hiMSA analysis compared to analyses using other methods and to hiMSA analyses of other acetylases (PDF)

S3 Fig hiMSA contrast hierarchical alignment of node-35 lineages. (PDF)

S4 Fig NAT root node alignment consisting of the consensus residues in each column position for each node in the hierarchy. The sequences used for the acetylase analysis are available at psed.igs.umaryland.edu.
(PDF)

S5 Fig Comparisons between BPPS-optimized and CDD-curated protein domain hierarchies. These figures were adapted from [23].
(PDF)
