# Supplementary material for: Inference of Functionally-Relevant N-acetyltransferase Residues Based on Statistical Correlations
Source: PLoS Comput Biol. 2016 Dec 21;12(12):e1005294. doi: 10.1371/journal.pcbi.1005294 (PMC5225019; doi:10.1371/journal.pcbi.1005294)
Supplement: S1 Fig — (PDF) [file pcbi.1005294.s001.pdf]

Inference of Functionally-Relevant N-Acetyltransferase Residues Based on Statistical Correlations

Andrew F. Neuwald and Stephen F. Altschul

S1_Figures. The hiHMM, hiMSA and full acetylase hierarchy.

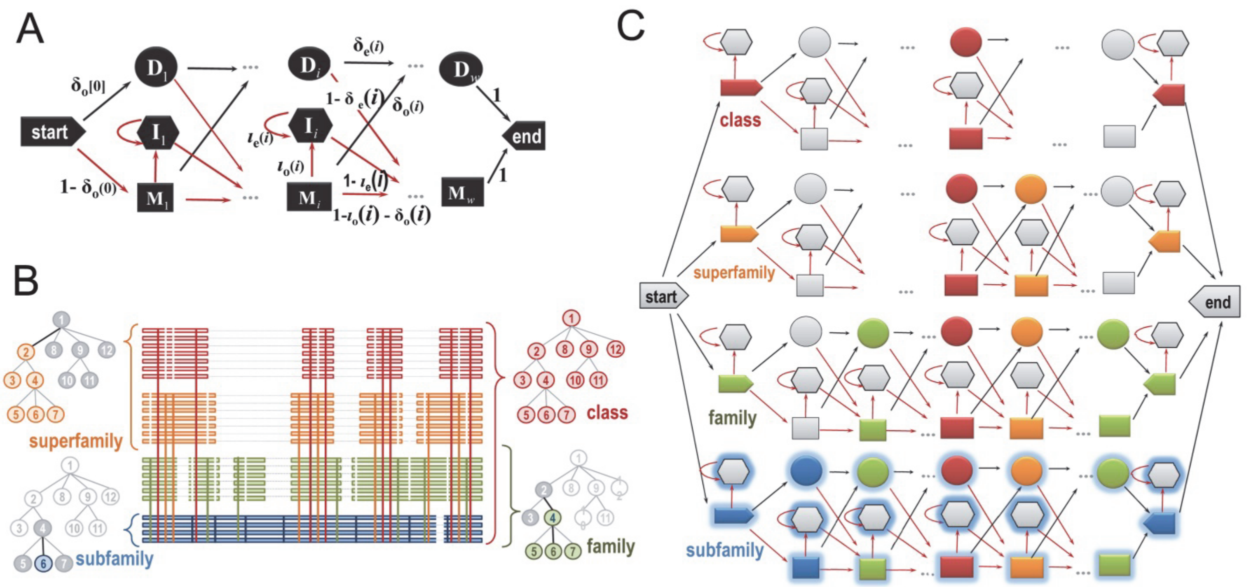

Figure S1.1. **A**. Architecture of a simple hidden Markov model (HMM) with position-specific transition probabilities. Red transition arrows emit a residue. **B**. Schematic diagram of a hiMSA from the perspective of a leaf node. One such diagram could be created for each node in a hierarchy. **(*center*)** The node 6 lineage of the full hiMSA. Horizontal lines represent aligned sequences and are color-coded by level in the hierarchy. Thin light gray horizontal lines represent non-homologous and deleted regions. Vertical lines represent the contrasting pattern positions upon which the hierarchy is based and are similarly color-coded by levels. **(*left & right sides*)** Subtrees corresponding to each level. The colored, gray and white nodes in each tree correspond, respectively, to their alignment foreground, background and non-participating partitions, the sequences of which are colored similarly. The background for the entire class **(*upper left*)** consists of random sequences. **C**. Features of a hiHMM from the perspective of the node 6 lineage in (B). Similarly colored HMM states share the same emission probability parameter settings. Such a hiHMMs are utilized by the hieraln program (see Methods).

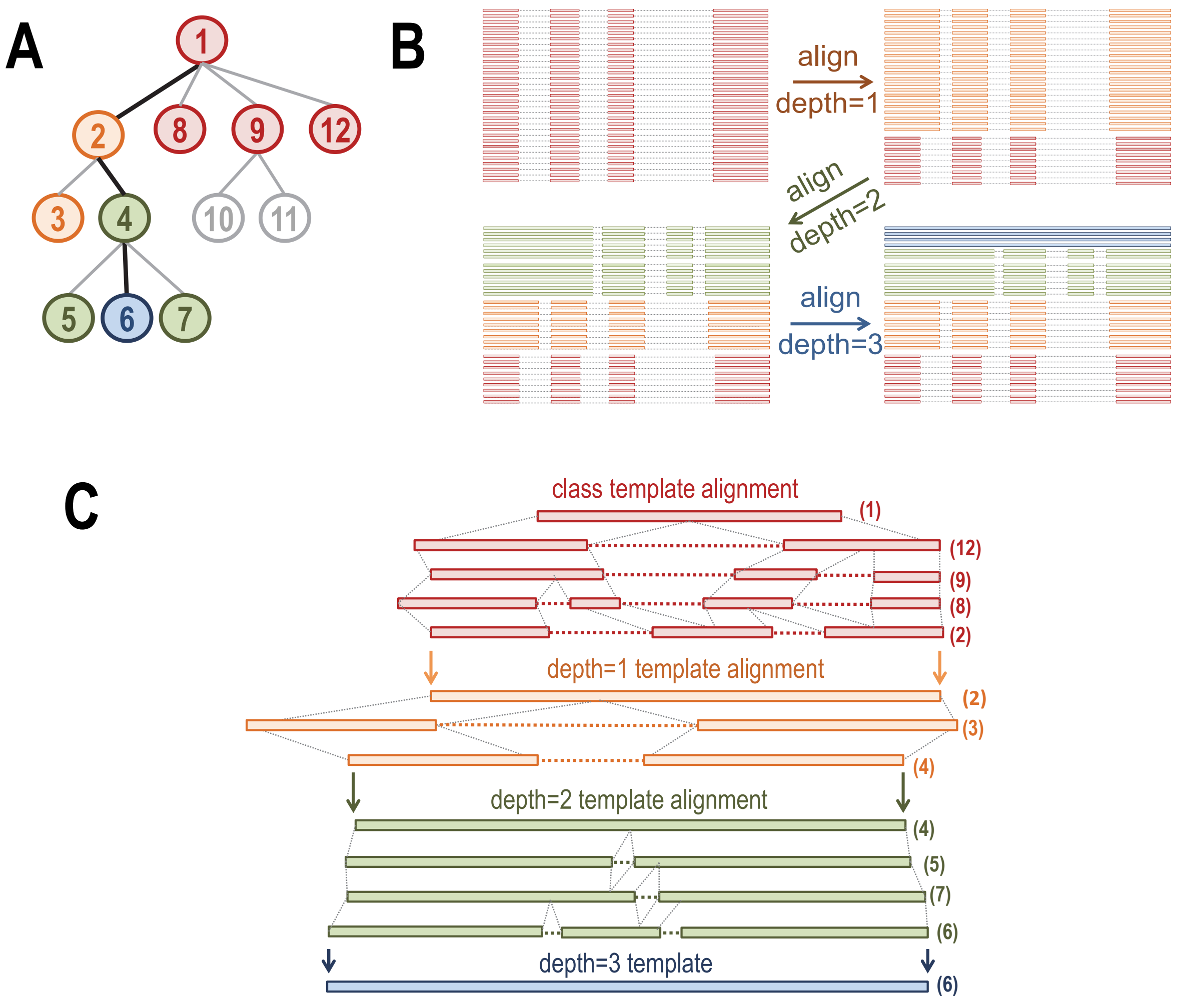

Figure S1.2. Expanding a hierarchical alignment into a hiMSA. **A**. Hypothetical hierarchy expanded in (B) and (C). **B**. Steps required to generate a lineage hierarchical alignment. Steps corresponding to the path from the root node to node 6 in (A): (a) The root alignment is hybridized with the subtree 2 alignment. (b) The previous alignment is hybridized with the subtree 4 alignment. (c) The alignment is hybridized with the node 6 alignment. **C**. Schematic of a template alignment for the lineage shown.

A

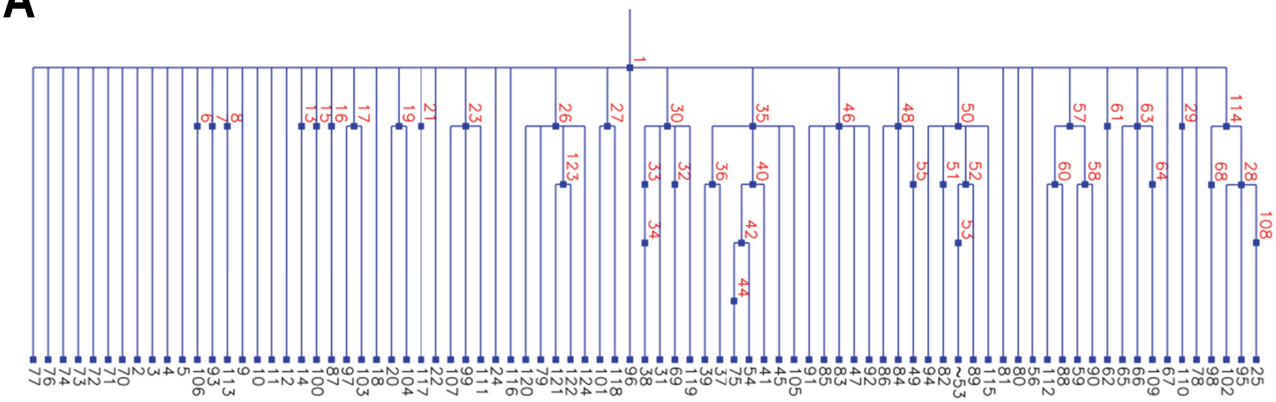

B

((((25)108,95,102)28,(98)68)114,78,(110)29,67,((109)64,66,65)63,(62)61
,((90,59)58,(88,112)60)57,56,80,81,(115,(89,(43)53)52,(82)51,94)50,((4
9)55,84,86)48,(92,47,83,85,91)46,(105,45,(41,(54,(75)44)42)40,(37,39)3
6)35,(119,(69)32,31,((38)34)33)30,96,(118,101)27,(124,(122,121)123,79,
120)26,116,24,(111,99,107)23,22,(117)21,(104,20)19,18,(103,97)17,(87)1
6,(100)15,(14)13,12,11,10,9,(113)8,(93)7,(106)6,5,4,3,2,70,71,72,73,74
,76,77)1;

Figure S1.3. The complete acetyltransferase hierarchy identified in this analysis. **A**. Tree representation. **B**. Newick representation.
