# Supplementary material for: Inference of Functionally-Relevant N-acetyltransferase Residues Based on Statistical Correlations
Source: PLoS Comput Biol. 2016 Dec 21;12(12):e1005294. doi: 10.1371/journal.pcbi.1005294 (PMC5225019; doi:10.1371/journal.pcbi.1005294)
Supplement: S2 Fig — (PDF) [file pcbi.1005294.s002.pdf]

Inference of Functionally-Relevant N-Acetyltransferase Residues Based on Statistical Correlations

Andrew F. Neuwald and Stephen F. Altschul

S2_Figures. Gna1 hiMSA analysis compared to analyses using other methods and to hiMSA analyses of other acetylases.

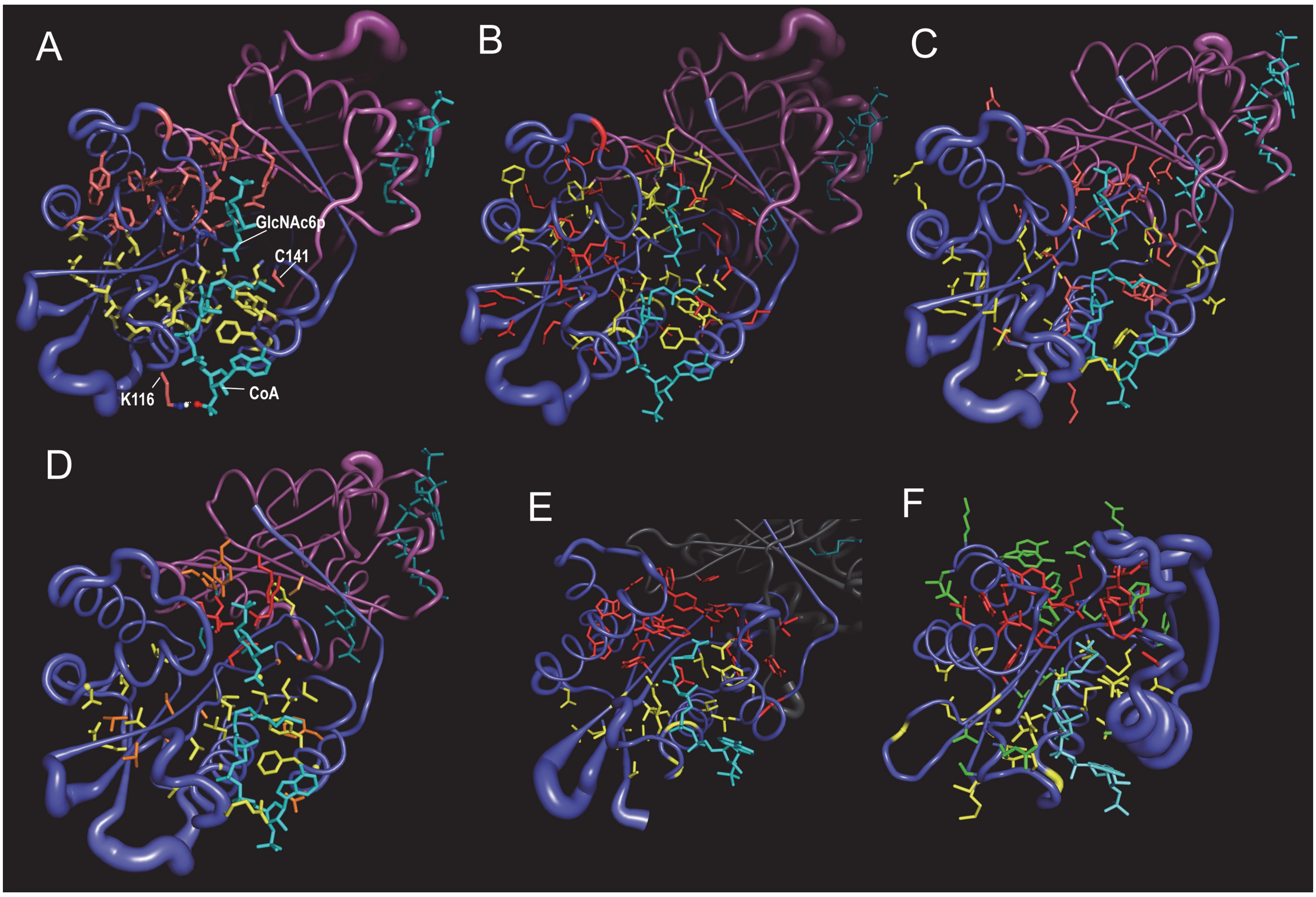

Figure S2.1. PyMol images of hiMSA analysis of Gna1 compared to analyses using other methods and to hiMSA analyses of other acetylases. **A**. hiMSA analysis of Gna1 (pdb_id 4ag9). Color scheme: the backbones of the two subunits within the homodimer, blue and violet; root node distinguishing residue sidechains, yellow; node 12 residues, red; CoA and substrate, cyan. **B**. Structural locations of Gna1 residues identified and classified by FRpred using Gna1 as a query and using default parameter settings. Color scheme: conserved residue sidechains, yellow; subtype residues, red. **C**. Gna1 residues classified by FRpred using an input alignment of 587 sequences assigned to node 12 (and sharing no more than 95% identity) and 2,073 randomly selected sequences among those assigned to the other non-root nodes of the **Fig 3A** hierarchy. (FRpred returned an error message on larger input alignments.) The color scheme is the same as in B. **D**. CLIPS-1D analysis of 4ag9 using the same input alignment as in C but with 50% of the sequences randomly selected and removed. (CLIPS-1D rejected larger input alignments.) Color scheme: structurally important residue sidechains, yellow; ligand-binding residues, orange; catalytic residues, red. **E**. hiMSA analysis of Hpa2 histone acetyltransferase (pdb_id: 1qsm); the Hpa2 subgroup is not shown in **Fig 3A**. Color scheme: the backbones of the two subunits within the homodimer, blue and dark gray; root node-specific residue sidechains, yellow; Hpa2-specific residues, red; CoA, cyan. **F**. hiMSA analysis of amino-terminal acetyltransferase catalytic subunit Naa10 (pdb_id: 4kvo); this protein was assigned to node 58 of the acetylase hierarchy in **Fig 3A**. Color scheme: backbone, blue; root node-specific residue sidechains, yellow; Node-57-specific residues, red; Naa10-specific residues, green; CoA, cyan.

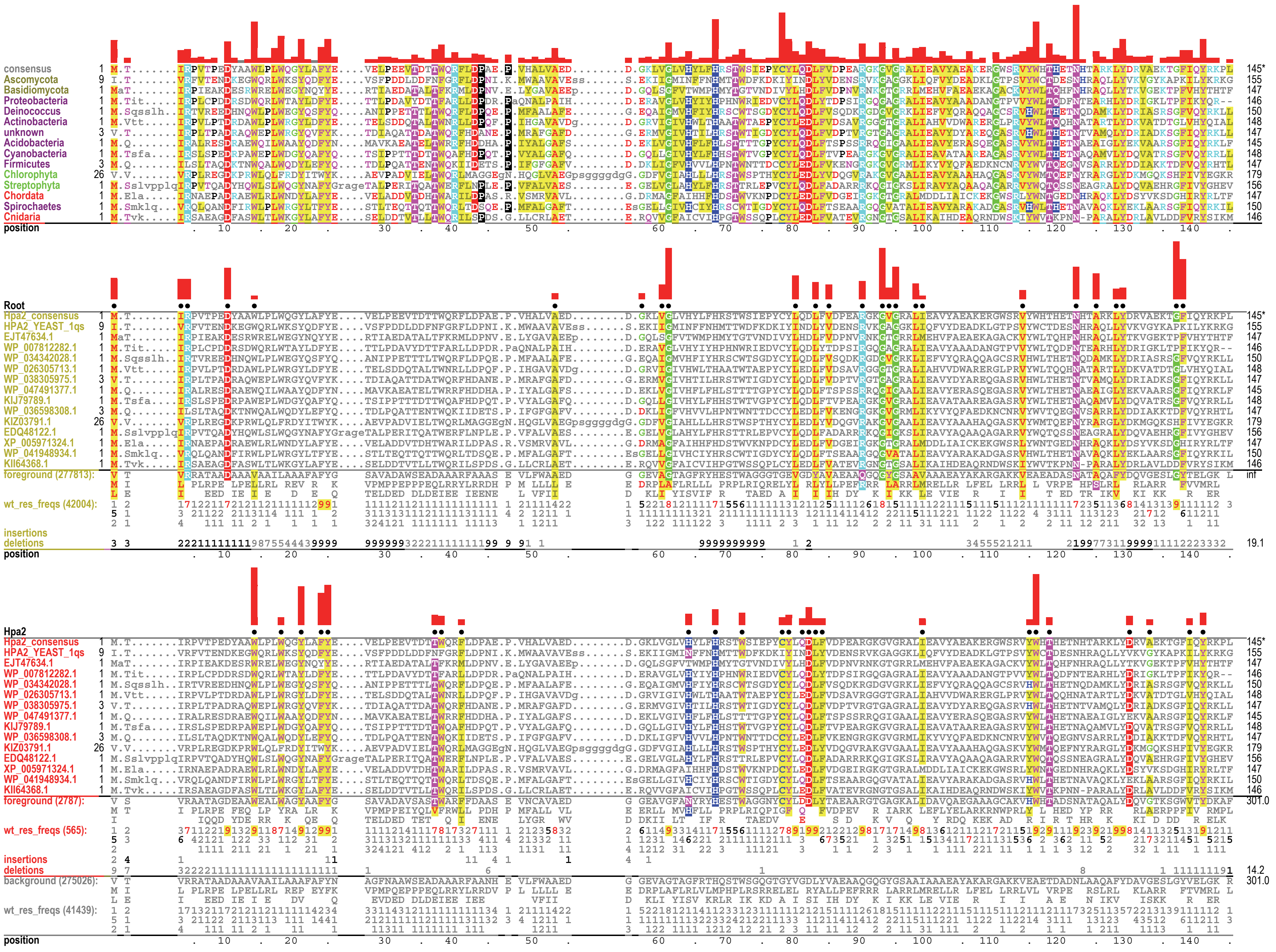

Figure S2.2. hiMSA contrast alignments for the Hpa2 histone acetyltransferase subgroup (node 56 in **Fig 3**). The foreground in the bottom alignment corresponds to the sequences assigned to the subtree rooted at node 56 and the background to the rest of the NAT hierarchy. The foreground for the second alignment corresponds to all acetylases and the background to non-acetylase sequences, represented by standard amino acid frequencies. The top alignment simply highlights all conserved positions in to the representative Naa10 sequences shown with bar heights indicated the degree of conservation; to the left the phylum for each sequence is named.

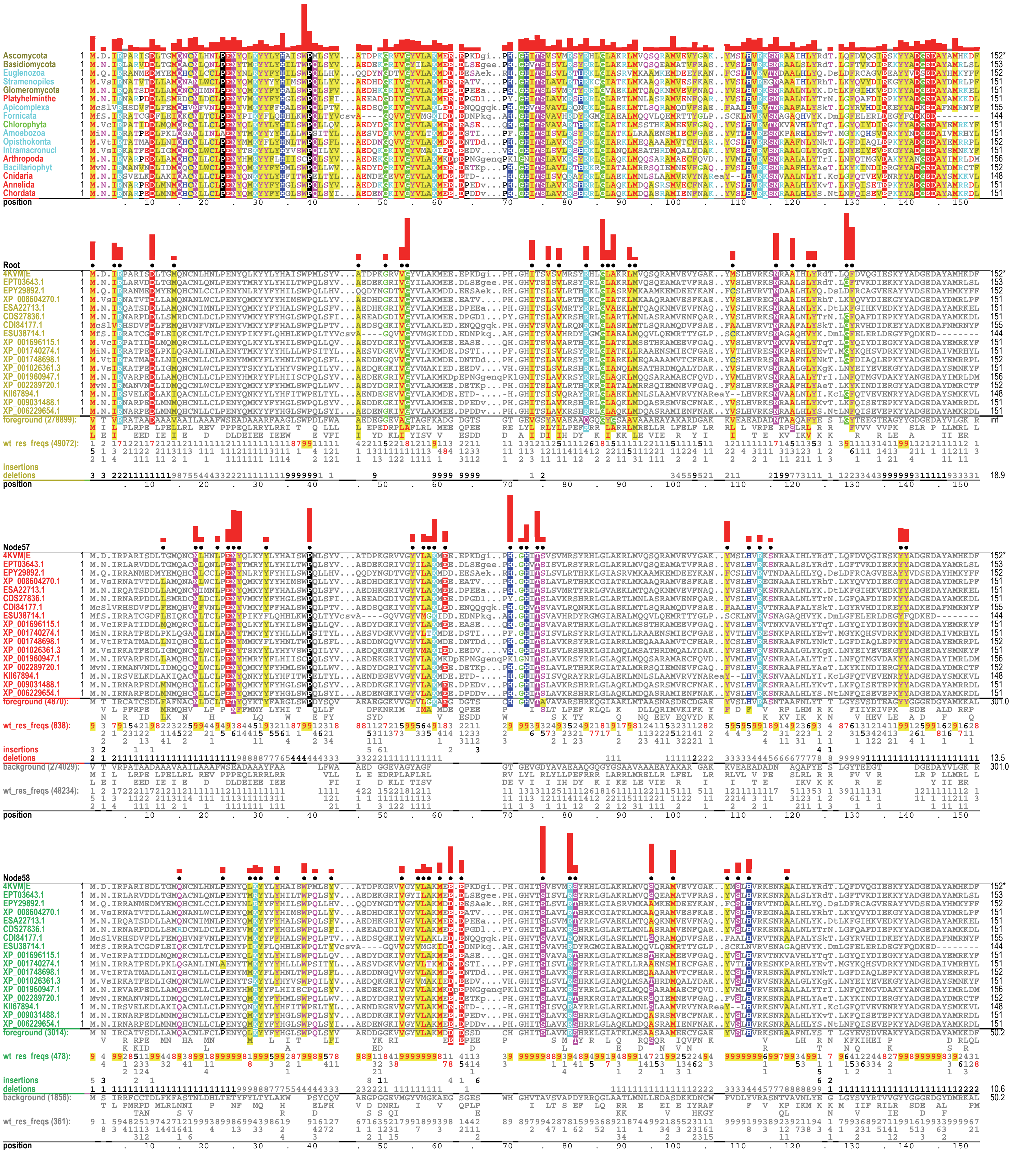

Figure S2.3. hiMSA contrast alignments for the amino-terminal acetyltransferase catalytic subunit Naa10 (node 58 in Fig 3A) subgroup. Note that the foreground in the bottom alignment corresponds to the sequences assigned to the subtree rooted at node 58 and the background to the rest of the sequences assigned to the parent of node 58, namely node 57. Likewise the foreground in the third alignment corresponds to the subtree rooted at node 57 and the background to the rest of the entire tree. The foreground for the second alignment corresponds to all acetylases and the background to non-acetylase sequences, represented by standard amino acid frequencies. The top alignment simply highlights all conserved positions in to the representative Naa10 sequences shown; to the left the phylum for each sequence is named.
