# Supplementary material for: Inference of Functionally-Relevant N-acetyltransferase Residues Based on Statistical Correlations
Source: PLoS Comput Biol. 2016 Dec 21;12(12):e1005294. doi: 10.1371/journal.pcbi.1005294 (PMC5225019; doi:10.1371/journal.pcbi.1005294)

Inference of Functionally-Relevant N-Acetyltransferase Residues Based on Statistical Correlations

Andrew F. Neuwald and Stephen F. Altschul

S3_Figure. hiMSA contrast hierarchical alignments for node-35 lineages.

A. Node Root to 35 lineage:

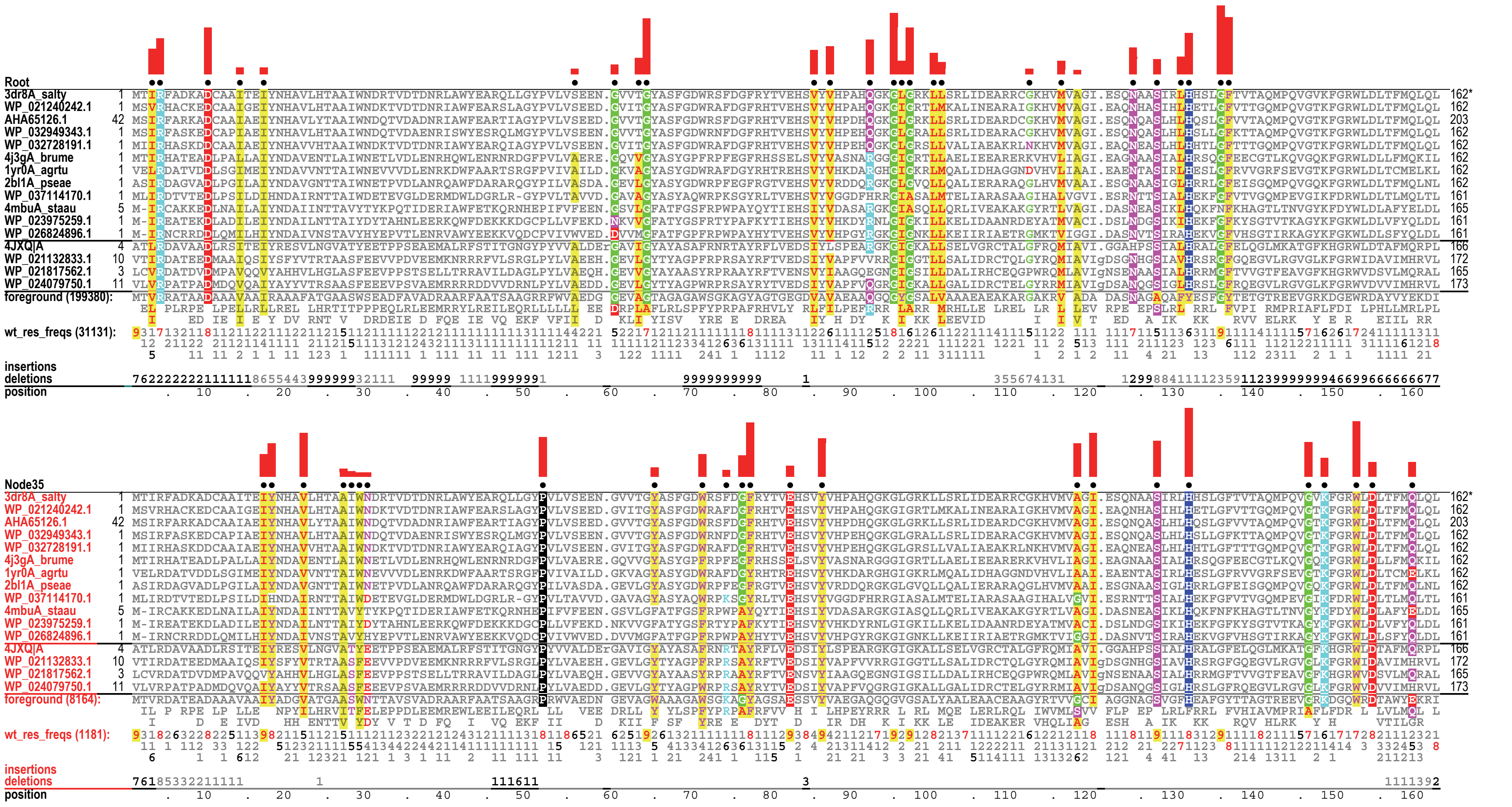

B. Node 40 to 42 lineage:

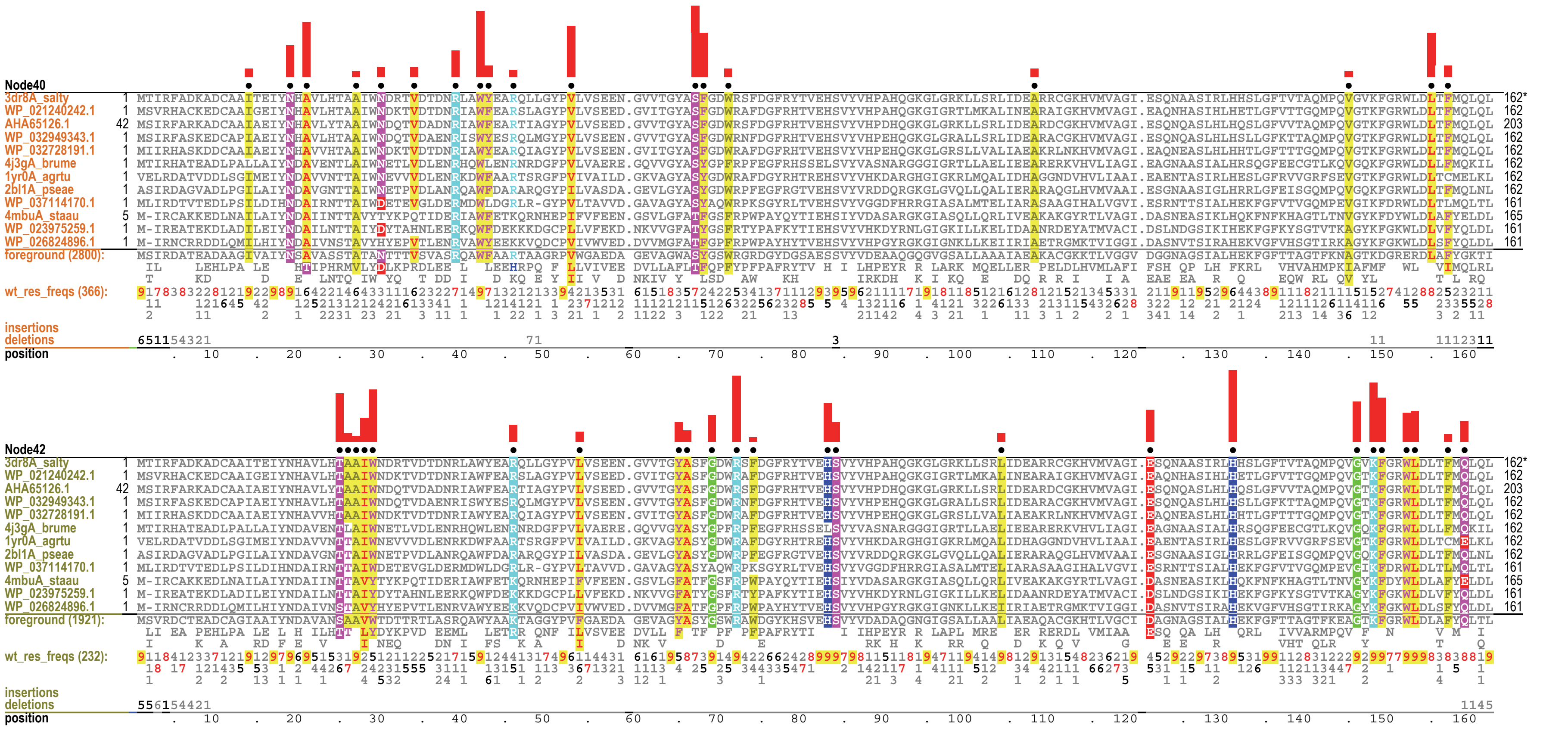

C. Node 36 to Node 39 lineage:

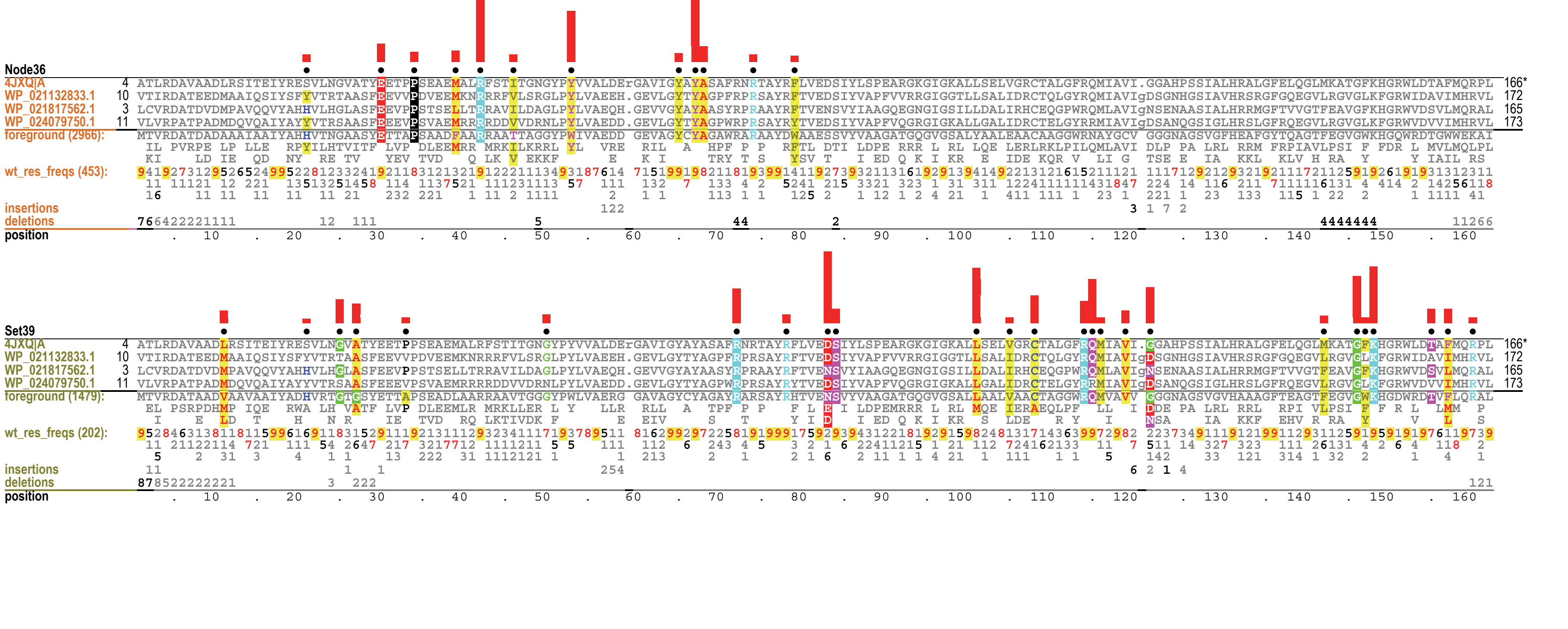

Supplement: S3 Fig — (PDF) [file pcbi.1005294.s003.pdf]
