# Supplementary material for: Inference of Functionally-Relevant N-acetyltransferase Residues Based on Statistical Correlations
Source: PLoS Comput Biol. 2016 Dec 21;12(12):e1005294. doi: 10.1371/journal.pcbi.1005294 (PMC5225019; doi:10.1371/journal.pcbi.1005294)

Inference of Functionally-Relevant N-Acetyltransferase Residues Based on Statistical Correlations

Andrew F. Neuwald and Stephen F. Altschul

Fig. S4. NAT root node alignment consisting of the consensus residues in each column position for each node in the hierarchy.

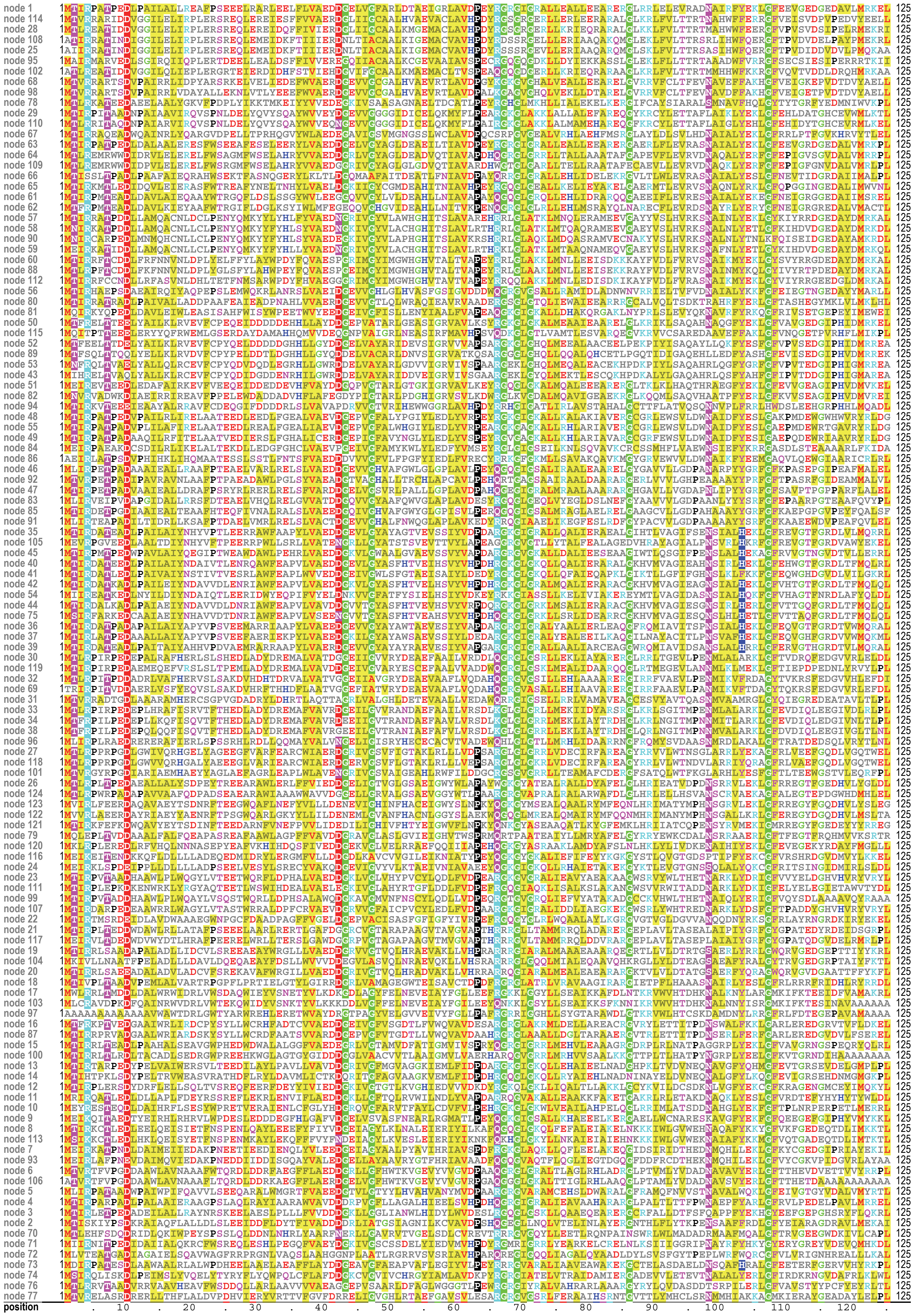

Supplement: S4 Fig — The sequences used for the acetylase analysis are available at psed.igs.umaryland.edu. (PDF) [file pcbi.1005294.s004.pdf]
