# Supplementary material for: Inference of Functionally-Relevant N-acetyltransferase Residues Based on Statistical Correlations
Source: PLoS Comput Biol. 2016 Dec 21;12(12):e1005294. doi: 10.1371/journal.pcbi.1005294 (PMC5225019; doi:10.1371/journal.pcbi.1005294)
Supplement: S5 Fig — These figures were adapted from [23]. (PDF) [file pcbi.1005294.s005.pdf]

Inference of Functionally-Relevant N-Acetyltransferase Residues Based on Statistical Correlations

Andrew F. Neuwald and Stephen F. Altschul

Figs. S5. Comparisons between BPPS-optimized and CDD-curated protein domain hierarchies. The methods used here are described in Neuwald, A.F. 2014. Evaluating, comparing and interpreting protein domain hierarchies. *Journal of Computational Biology* 21(4): 287-302; the link to this article is https://www.ncbi.nlm.nih.gov/pmc/articles/PMC3962652/ and to the programs for performing these comparisons is http://chain.umaryland.edu/omcbpps/ .

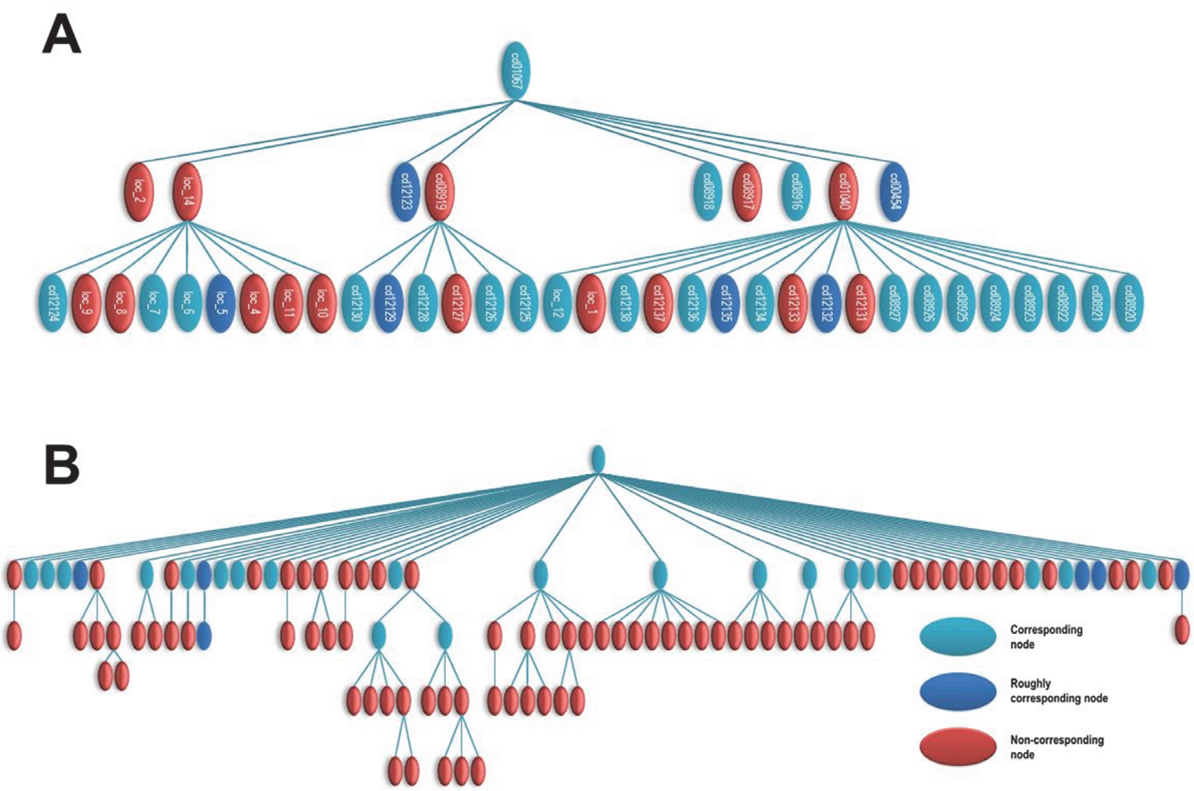

Fig. S5.1. Comparison of a Conserved Domain Database (CDD) hierarchy curated by the NCBI with a BPPS-optimized version of the same hierarchy. Nodes in blue are shared by both hierarchies whereas nodes in red are distinct between the two hierarchies. Note that the sequences assigned to each subtree rooted at a given node are implicitly assigned to that node as well (i.e., if a sequence belongs to a subfamily within a family it belongs to the family as well). Thus a leaf node in hierarchy A, for example, may correspond to a subtree in hierarchy B, in which case only the root node of the subtree in B will correspond to the leaf node in A. **A**. The globin-like CDD hierarchy (cd01067). **B**. The same globin-like hierarchy after BPPS-optimization, which removed some nodes and added others.

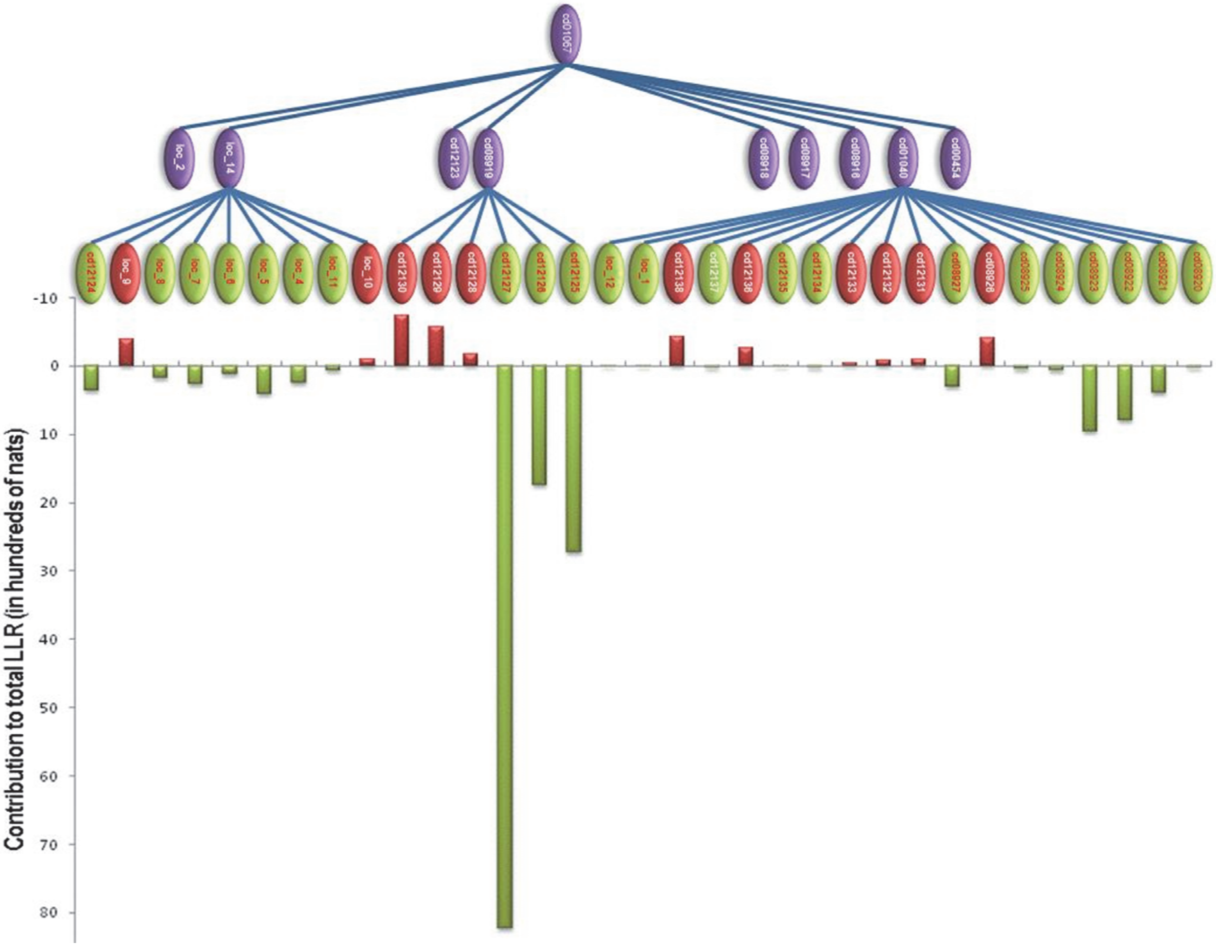

Fig. S5.2. Statistical support for depth 2 nodes within the CDD-curated globin-like hierarchy shown in Fig. S5.1. Depth 2 nodes in green have some (albeit in many cases weak) statistical support whereas those in red lack such support based on their contributions to the overall log-likelihood ratio (LLR). The average LLR contribution for positively contributing nodes was 516 nats (267 not including the maximum).

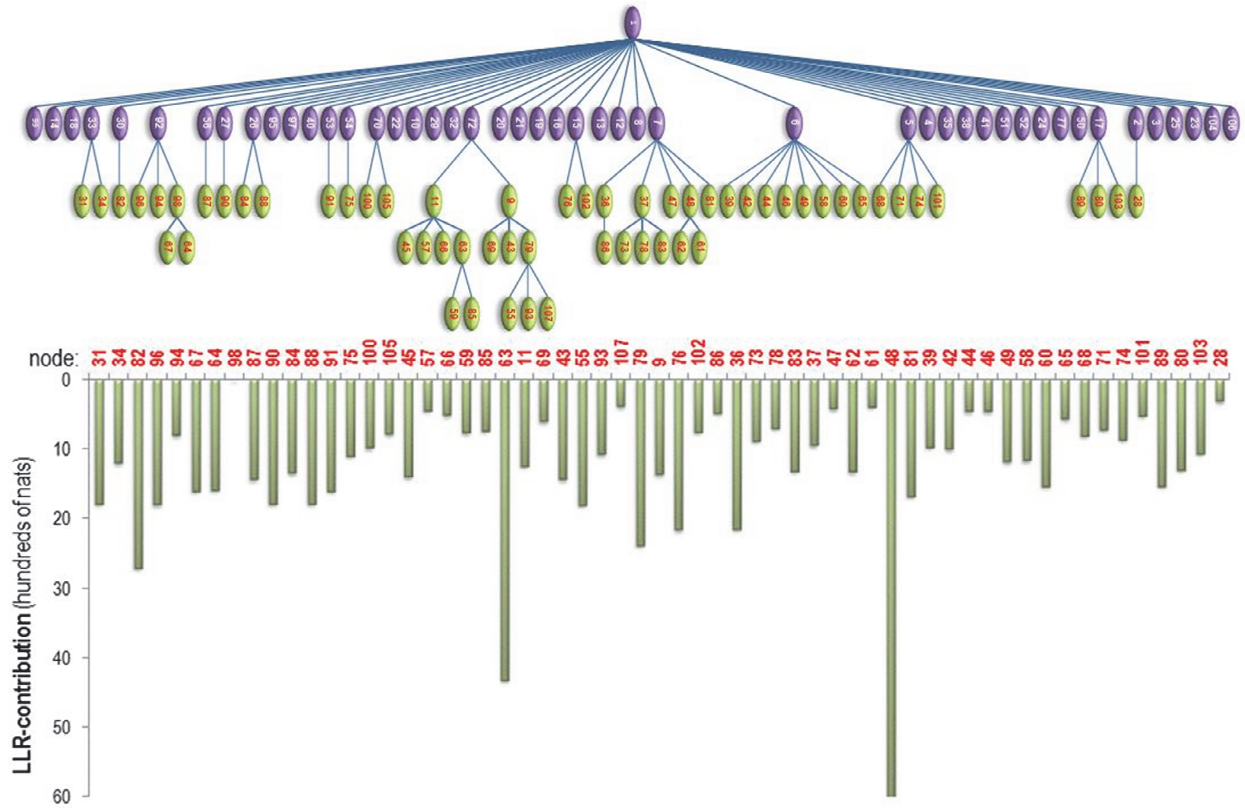

Fig. S5.3. Statistical support for depth ≥2 nodes within the BPPS-optimized globin-like hierarchy shown in Fig. S5.1. Nodes in green have statistical support based on their contributions to the overall log-likelihood ratio (LLR). On average the level of support is 1,263 nats; much higher than that observed for the globin-like CDD hierarchy in Fig. S5.2.
